# Sea-ice melt determines seasonal phytoplankton dynamics and delimits the habitat of temperate Atlantic taxa as the Arctic Ocean atlantifies

**DOI:** 10.1093/ismeco/ycae027

**Published:** 2024-02-27

**Authors:** Ellen Oldenburg, Ovidiu Popa, Matthias Wietz, Wilken-Jon von Appen, Sinhue Torres-Valdes, Christina Bienhold, Oliver Ebenhöh, Katja Metfies

**Affiliations:** Institute of Quantitative and Theoretical Biology, Heinrich-Heine-University Düsseldorf, Universitätsstr. 1, D-40225 Düsseldorf, Germany; Institute of Quantitative and Theoretical Biology, Heinrich-Heine-University Düsseldorf, Universitätsstr. 1, D-40225 Düsseldorf, Germany; Max Planck Institute for Marine Microbiology, Celsiusstraße 1 D-28359 Bremen, Germany; Deep-Sea Ecology and Technology, Alfred Wegener Institute Helmholtz Centre for Polar and Marine Research, Am Handelshafen 12 D-27570 Bremerhaven, Germany; Physical Oceanography of the Polar Seas, Alfred Wegener Institute Helmholtz Centre for Polar and Marine Research, Am Handelshafen 12 D-27570 Bremerhaven, Germany; Physical Oceanography of the Polar Seas, Alfred Wegener Institute Helmholtz Centre for Polar and Marine Research, Am Handelshafen 12 D-27570 Bremerhaven, Germany; Max Planck Institute for Marine Microbiology, Celsiusstraße 1 D-28359 Bremen, Germany; Deep-Sea Ecology and Technology, Alfred Wegener Institute Helmholtz Centre for Polar and Marine Research, Am Handelshafen 12 D-27570 Bremerhaven, Germany; Institute of Quantitative and Theoretical Biology, Heinrich-Heine-University Düsseldorf, Universitätsstr. 1, D-40225 Düsseldorf, Germany; Polar Biological Oceanography, Alfred Wegener Institute Helmholtz Centre for Polar and Marine Research, Am Handelshafen 12 D-27570 Bremerhaven, Germany

**Keywords:** climate change, time-series clustering, marine, Arctic aquatic communities, Atlantification, Fourier decomposition

## Abstract

The Arctic Ocean is one of the regions where anthropogenic environmental change is progressing most rapidly and drastically. The impact of rising temperatures and decreasing sea ice on Arctic marine microbial communities is yet not well understood. Microbes form the basis of food webs in the Arctic Ocean, providing energy for larger organisms. Previous studies have shown that Atlantic taxa associated with low light are robust to more polar conditions. We compared to which extent sea ice melt influences light-associated phytoplankton dynamics and biodiversity over two years at two mooring locations in the Fram Strait. One mooring is deployed in pure Atlantic water, and the second in the intermittently ice-covered Marginal Ice Zone. Time-series analysis of amplicon sequence variants abundance over a 2-year period, allowed us to identify communities of co-occurring taxa that exhibit similar patterns throughout the annual cycle. We then examined how alterations in environmental conditions affect the prevalence of species. During high abundance periods of diatoms, polar phytoplankton populations dominated, while temperate taxa were weakly represented. Furthermore, we found that polar pelagic and ice-associated taxa, such as *Fragilariopsis cylindrus* and *Melosira arctica*, were more common in Atlantic conditions, while temperate taxa, such as *Odontella aurita* and *Proboscia alata*, were less abundant under polar conditions. This suggests that sea ice melt may act as a barrier to the northward expansion of temperate phytoplankton, preventing their dominance in regions still strongly influenced by polar conditions. Our findings highlight the complex interactions between sea ice melt, phytoplankton dynamics, and biodiversity in the Arctic.

## Introduction

**Figure 1:**
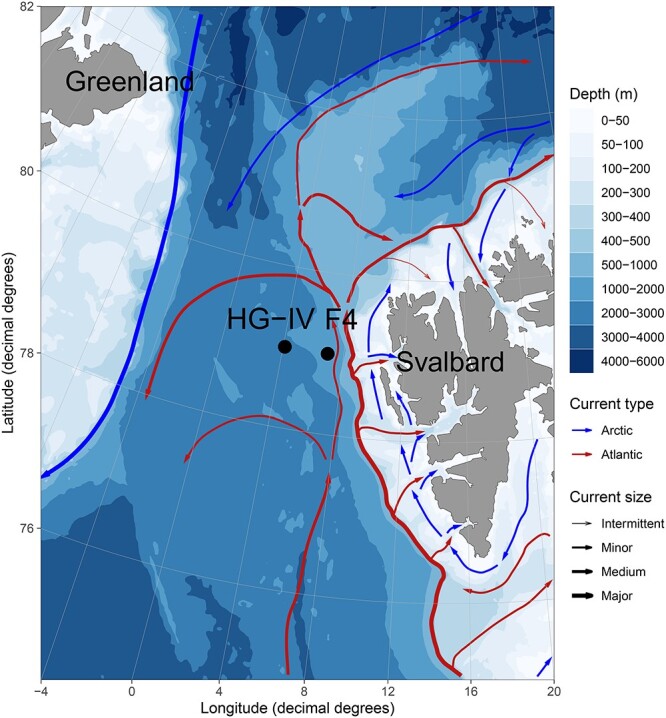
**Map of mooring locations, major currents, and water depths in Fram Strait**. The main currents in the area are illustrated schematically: West Spitsbergen Current (WSC) in red and East Greenland Current (EGC) in blue. The locations of the moored remote access samplers discussed in this study are marked in black for HG-IV and F4. F4 is located in the WSC and HG-IV west of the WSC. Land is displayed in gray and the different water depths in a white-blue color gradient.

The Arctic is affected by rapid and drastic environmental changes. For instance, air temperatures rise four times [1] as quickly in the region compared to other regions on Earth [2]. Arctic sea ice is one of the fastest changing components of the Earth system [3]. Over the past decades, the area of Arctic sea ice declined at a rate of ~1 million km^2^ in area extent per decade [3, 4]. There are indications for a 40% decline in ice thickness due to thicker and older ice cover [5]. The geographical extent of warmer and more saline Atlantic water is expected to expand northwards into the Central Arctic Ocean (CAO), which consequently will become warmer and saltier, further accelerating sea-ice decline [6]. This process, called Atlantification of the Arctic Ocean [6], coincides with altered physical conditions. Ecosystems shift towards a more temperate state including the appearance and range expansion of subarctic specie [7-12]. If the temperature increases and the loss of sea-ice continue at their current pace, the Arctic Ocean will likely be seasonally ice-free by 2050 [13]. In such a scenario, sea-ice melt-related processes, such as melt-water stratification of the upper layer of the ocean, that is currently observed in the marginal ice zone (MIZ), might become more important over more prolonged periods throughout the seasonal cycle, and a larger geographic area, with ecological consequences for the Arctic Ocean. The MIZ is usually covered with 15–80% sea ice [14-20] and its distribution, thickness, and melt dynamics are key drivers of productivity [21], carbon export, biogeochemical cycling, and pelagic-benthic coupling. As a result of decreasing sea ice extent and the expected Atlantification, larger areas of the Arctic Ocean might become favorable for pelagic temperate phytoplankton. As a study site, Fram Strait allows us to investigate the combined effects of Atlantification and seasonal ice cover on Arctic marine ecosystems. Moorings with a suite of physical and biogeochemical sensors, as well as autonomous sampling systems for molecular biodiversity studies (Remote Access Sampler RAS), are positioned at two different locations in Atlantic Waters of Fram Strait at ∼79°N: central Fram Strait (mooring cluster “HG-IV”) and in the eastern Fram Strait (mooring cluster “F4”) -see Figure 1. F4 is located in the flow path of the West Spitsbergen Current (WSC). HG-IV is located in the vicinity of the interface between the WSC and the East Greenland Current (EGC). The WSC carries relatively warm and salty Atlantic Water via Fram Strait northwards towards the CAO, while the EGC exports cold ice-covered and less saline Polar Water (PW) from the CAO through Fram Strait. In the vicinity of HG-IV, some of the Atlantic Water (AW) is mixed in an eddy-rich area [22] as part of a subduction process [23, 24] with the outflowing colder and fresher water of the EGC. This area is frequently characterized by major sea-ice melt events, as sea-ice coverage regularly extends [25] into the WSC, which carries temperate species towards the CAO. Thus, ecosystem functionality in the vicinity of the MIZ in the WSC might serve as a model for future biodiversity and ecosystem functionality in a seasonally ice-free CAO impacted by Atlantification and thereby inform on the potential of temperate taxa to thrive in a seasonally ice-covered Atlantic-influenced Ocean [8, 26-28].

Over the past few decades, the transport of sea ice in both volume and velocity towards Fram Strait increased in the area of the Transpolar Drift due to the thinning Arctic pack ice [29-31]. This led to a significant south-eastward extension of the MIZ into Fram Strait during certain years of the past decade. In 2017, the MIZ extended into large parts of the WSC during summer, including the two moorings [31]. Conversely, the 2018 ice export was reduced to <40% relative to that between 2000 and 2017.

The associated meltwater-induced stratification promoted a longer phytoplankton bloom with a relatively shallow extent and reduced export flux [32]. The summer of 2018 had a mixed layer regime (MLR) and a shorter, more intense bloom compared to other periods. During the spring of that year, there was also an increased carbon export to the deep sea [33]. The particularly warm year of 2018 may reflect the conditions of the CAO in the future. The native biodiversity of the communities is a key determinant of whether and how a community or an individual organism can respond to changing abiotic conditions [34]. We, therefore, expect that studying the microbial communities and, in particular, comparing the seasonal dynamics between the years 2017 and 2018 can greatly improve our knowledge about the resilience of pelagic and sympagic organisms and how microbial diversity and seasonality scale with the environmental variability. Molecular biodiversity research using ribosomal meta-barcoding has substantially improved our comprehension of marine microbial diversity and distribution patterns during the last 20 years. [35, 36]. As part of the FRAM Infrastructure Program (Frontiers in Arctic Marine Monitoring) and the long-term ecological research site LTER HAUSGARTEN, activities in Fram Strait provide information on Arctic marine eukaryotic microbial biodiversity and biogeography based on annually recurring measurements (since 1999) recently expanded by year-round, continuous sampling since 2016. We hypothesize that biodiversity and seasonal succession in the Fram Strait are strongly impacted by sea-ice melt and the extent of stratification [37].

In this study, we exploit this wealth of data through a combination of statistical and bioinformatic approaches. The continuous data collected over two years were decomposed using a Fourier transformation into a series of sinusoidal functions. Each function represents a specific amplicon sequence variant (ASV) dynamic over time. By clustering the ASVs based on their seasonal fluctuation patterns, it became possible to analyze the impact of different water regimes that occurred in 2017 and 2018, as reported in Appen et al. 2021 [32], on both species and community levels. We could elucidate the effects of sea-ice melt on the seasonal dynamics of the associated eukaryotic microbial communities as key drivers of phytoplankton bloom phenology. By assessing the contribution of polar and temperate phytoplankton taxa to eukaryotic microbial communities in the WSC over the annual cycle, we infer the potential of polar taxa to thrive in ice-free Atlantic water and temperate taxa to expand to areas impacted by sea-ice melt.

## Materials and methods

### Sampling

The samples analyzed in this study were collected using McLane Remote Access Samplers (RAS) deployed in conjunction with other oceanographic sensors over three individual annual cycles from June 2016–August 2019 on long-term moorings at stations HG-IV (79.0118 N 4.1666E) and F4 (79.0118 N 6.9648E) of the LTER HAUSGARTEN and FRAM in the Fram Strait [38]. This study covers the period from January 2017 to December 2018, i.e., two calendar years. One RAS was deployed at a depth between 24–29 m at HG-IV and another at 23-26 m - at F4. The RAS samplers contained 48 sterile bags, each collecting water samples of 500 mL at programmed sampling events every two weeks. Samples were preserved by adding 700 μl of half-saturated mercuric chloride (7.5% w/v) to the bags prior to sampling. A sample reflects the pool of up to two samples collected one hour apart in two individual bags. Following the recovery of the RAS devices, water samples were filtered using Sterivex filter cartridges with a pore size of 0.22 μm (Millipore, USA). Filters were then stored at −20°C for later processing.

### Mooring and satellite data

Temperature, salinity, and dissolved oxygen concentration were measured with a CTD-O_2 attached to the RAS frame. Physical oceanography sensors were manufacturer-calibrated and processed as described in [39]. Raw and processed mooring data are available at PANGAEA https://doi.org/10.1594/PANGAEA.904565, https://doi.org/10.1594/PANGAEA.940744, https://doi.pangaea.de/10.1594/PANGAEA.941125. For chemical sensors, raw sensor readouts were used. The fraction of Atlantic and Polar Water were computed for each sampling event following [23] and reported along with distance below the surface (due to mooring blowdown). Sea ice concentration derived from the Advanced Microwave Scanning Radiometer sensor AMSR-2 [40] were downloaded from the Institute of Environmental Physics, University of Bremen (https://seaice.uni-bremen.de/sea-ice-concentration-amsr-eamsr2). Sentinel 3A OLCI chlorophyll surface concentrations were downloaded from https://earth.esa.int/web/sentinel/sentinel-data-access. For all satellite-derived data, we considered grid points within a radius of 15 km around the moorings. Similar to van Appen et al. 2021 [41], the analyzed datasets consist of ten environmental values for the two locations, F4 and HG-IV, from 01.01.2017 to 31.12.2018. From this dataset, we retrieved the following variables: water temperature (temp °C), fluorescence chlorophyll concentration from in situ sensor (chl_sens ∼*μ*g *l*^*−*1^), daylight (daylight h), water depth (depth m), ice concentration (iceConc %), ice distance (IceDist to 20% ice concentration km), mixed layer depth (MLD m), partial pressure of C*O*_2_ (pC*O*_2__conc *μ*atm), *O*_2_ concentration (O_2_ _conc *μ*mol *l*^*−*1^), polar-water fraction (PW_frac %).

### DNA-extraction and Illumina amplicon-sequencing of 18S rRNA genes

Isolation of genomic DNA was carried out using the PowerWater kit (Qiagen, Germany) following the manufacturer’s protocol. Obtained DNA was quantified using Quantus (Promega, USA) and stored at −20°C. 18S rRNA gene fragments from the hypervariable V4 region were amplified by polymerase chain reaction (PCR) with primers 528iF (GCGGTAATTCCAGCTCCAA) and 926iR (ACTTTCGTTCTTGATYRR). illuminaNextV4F (TCGTCGGCA GCGTCAGATGTGTATAAGAGACAGGCGGTAATTCCAGCTCC) and illuminaNextV4R (GTCTCGTGGGCTCG-GAGATGTGTATAAGAGACAGGGCAAATGCTTTCGC) [42]. All PCRs had a final volume of 50 μL and contained 0.02 U Phusion Polymerase (Thermo Fisher, Germany), the 10-fold polymerase buffer according to manufacturer’s specification, 0.8 mM each dNTP (Eppendorf, Germany), 0.2 μM *L*^*−*1^ of each primer, and 1 μL of template DNA. PCR amplification was performed in a thermal cycler (Eppendorf, Germany) with an initial denaturation (94°C, 2 min) followed by 35 cycles of denaturation (94°C, 20 sec), annealing (58°C, 30 sec), and extension (68°C, 30 sec) with a single final extension (68°C, 10 min). The PCR products were purified from an agarose gel 1% [w/v] with the NucleoSpin Gel Kit (Macherey-Nagel, Germany) and Mini Elute PCR Purification kit (Qiagen, Germany). Subsequently, DNA concentrations were determined using a Quantus Fluorometer (Promega, USA). Prior to library preparation, DNA fragments were diluted with TE buffer to a concentration of 0.2 ng μL^−1^. Libraries were prepared according to the 16S Metagenomic Sequencing Library Preparation protocol, and sequenced using MiSeq (Illumina, USA) in 2x300 paired-end runs. Sequence data are available under ENA BioProjects PRJEB43889 and PRJEB43890.

### Sequence analysis

After primer removal using cutadapt [43], reads were processed into amplicon sequence variants (ASVs) using DADA2 v1.14.1 [39], as described in Wietz et al [44]. Briefly, reads were trimmed based on quality profiles, with filtering settings truncLen = c(250, 200), maxN = 0, minQ = 2, maxEE = *c*(3*,* 3), and trunc*Q* = 0. Followed by merging (minOverlap = 20) and chimera removal, reads were taxonomically classified using PR2 v4.12 [45]. The herein reported data has been processed in the scope of autonomous eDNA biodiversity analyses within the FRAM Observatory, as described under https://github.com/matthiaswietz/FRAM-RAS_eDNA.

### Analysis strategy and R packages

All calculations were performed in R version 4.1.3 (2022-03-10). The complete analysis pipeline is available at https://gitlab.com/qtb-hhu/qtb-sda/framstrait_1718. Analysis and plotting tools used for this work are available in a git repository with scripts and an R package. Fourier decomposition was performed with the segmenTier R package [46], available at https://cran.r-project.org/package=segmenTier. The dynamics of eukaryotes were analyzed using the Fourier-transformed time series signals of the relative abundance information. As part of biodiversity, relative species abundance refers to the extent to which a species is common or rare relative to other species in a particular location or community [47]. Relative abundance is the percentage composition of an organism of a given species relative to the total number of organisms in that habitat. The data were interpolated on daily bases.

### Time series analysis

The use of Fourier decomposition for time series signals is a common technique to obtain temporal profiles of data that contain seasonal patterns. In this study, we used this technique to identify and describe the seasonality of several species, as also described in Priest et al. [48]. For each amplicon sequence variant we extracted the time series signal from the relative abundance data using a Fourier approach implemented in the R package segmenTier / segmenTools [49]. The Fourier technique is decomposing signals into the sum of their frequency components, characterized by sine and cosine functions. The Fourier Theorem states that any function can be rewritten as the sum of sinusoidal functions. The approximation becomes more accurate with each additional series element. These elements are called Fourier components.

A measurement for seasonality s for the times series t was calculated by the following formula:


(1) \begin{equation*}s(t) = \frac{\lvert f_2(t)\rvert}{\lvert f_0(t)\rvert}, \end{equation*}

where f_i_ is the i-th fourier component of the times series t and |⋅| is the absolute value function [50, 51].

After the Fourier transformation, the frequency, amplitude, and phase information of each particular ASV time signal was extracted. These values indicate the seasonality, abundance strength, and time of occurrence within the measured period.

**Cluster definition**

Species with similar temporal pattern were grouped into co-occurrence clusters. The choice for the parameter N = 10, the number of clusters for both locations, was chosen to keep the cluster comparable. The metric (Bayesian Information Criterion - BIC) of the applied clustering algorithm proposes a value around 9 and 10 as the optimal cluster number. Groups of species with similar time signals were identified by a clustering approach in the segmenTools R package [49]. The significance of overlapping clusters (shared members by two clusters), illustrated as a color gradient, is calculated based on the negative logarithm of the p-value and the number of overlapping features. All identified clusters were classified into low-light, high-light, and mixed-light clusters depending on the light conditions in which their members show the highest abundance. Further, all clusters were named depending on the mooring (H for HG-IV and F for F4) and numbered in ascending order depending on the phase of the sinusoidal function, which was calculated for each cluster from the average of the cluster members. Therefore, the order of the numbers indicates the order of occurrence within the year.

### Co-occurrence of ASVs

In contrast to earlier investigations that depended on Pearson correlation for pairwise comparisons of relative abundance values to deduce co-occurrence patterns our methodology utilized Fourier decomposition of time series data [52-54]. This allowed the extraction of unique temporal profiles for each Amplicon Sequence Variant (ASV). By applying correlation analysis to these individual profiles, we effectively mitigated the inherent bias associated with utilizing Pearson correlation on compositional data [55] .

### Conditions preference

To assess the population’s annual abundance, we computed the sum of relative abundances for each Amplicon Sequence Variant (ASV) within a specified timeframe. Total abundance values were separately calculated for the F4 and HG-IV locations. Subsequently, entries with zero abundance were excluded to prevent division by zero, and we determined the abundance quotients for 2017 and 2018, as well as the reverse calculation. The log2(quotient) values were categorized as meltwater regime (MWR) or MLR based on whether they were greater than or equal to 1 or less than or equal to −1, respectively. ASVs not meeting either condition were assigned to the unspecified group. To gauge the dissimilarity between locations in a given year for a specific group of ASVs, we defined four quotients as follows:


(2) \begin{equation*} p\left(x,y\right)=\frac{{\left.\mathrm{MWR}\right|}_x}{{\left.\mathrm{MWR}\right|}_y},\kern2em x\in X,y\in Y, \end{equation*}

(3) \begin{equation*} t\left(x,y\right)=\frac{{\left.\mathrm{MLR}\right|}_x}{{\left.\mathrm{MLR}\right|}_y},\kern2em x\in X,y\in Y, \end{equation*}

where X = {F417, F418}, Y = {HG-IV17, HG-IV18}, and MWR (MLR) containing all MWR (MLR) ASVs relative two-year abundances. The restriction is defined by selecting only the ASV abundances from the given time and location.

- p(F42017, HG-IV2017) correspond to the ratio of F4 to HG-IV for species preferring the MWR in 2017.
- p(F42018, HG-IV2018) correspond to the ratio of F4 to HG-IV for species preferring the MWR in 2018.
- t(F42017, HG-IV2017) correspond to the ratio of F4 to HG-IV for species preferring the MLR in 2017.
- t(F42018, HG-IV2018) correspond to the ratio of F4 to HG-IV for species preferring the MLR in 2018.

To compare how much the MWR is favoured on average versus a MLR within a given site, we define the following equations:


(4) \begin{equation*} q(z)=\frac{\frac{1}{\left|\mathrm{MWR}\right|\left.{}_z\right|}{\sum}_{i\in{\left.\mathrm{MWR}\right|}_zi}{\left.\mathrm{MWR}\right|}_z}{\frac{1}{\left|\mathrm{MLR}\right|\left.{}_z\right|}{\sum}_{h\in{\left.\mathrm{MLR}\right|}_zh}{\left.\mathrm{MLR}\right|}_z},\kern2em z\in Z, \end{equation*}

where Z = {F417, F418, HG-IV17, HG-IV18}, and MWR (MLR) containing all MWR (MLR) ASVs relative two-year abundances. The restriction is defined by selecting only the ASV abundances from the given time and location and _i_MWR (_h_MLR) is the i-th (h-th) relative two-year abundance from the MWR (MLR) ASV.

- q(F42017) corresponds to the ratio for meltwater preference over mixed-layer in 2017 at station F4.
- q(F42018) corresponds to the ratio for meltwater preference over mixed-layer in 2018 at station F4.
- q(HG-IV2017) corresponds to the ratio for meltwater preference over mixed-layer in 2017 at station HG-IV.
- q(HG-IV2018) corresponds to the ratio for meltwater preference over mixed-layer in 2018 at station HG-IV.

### Cross-condition analysis

To investigate how the dynamics of a particular ASV with a preference for a specific water regime change under the conditions of the opposite water regime, we determined and compared the area under the curve (AUC) from the relative abundance within a time range of 365 days. For that, we used on a daily level interpolated abundance data to which we applied a polynomial function and calculated the AUC for each year separately. Afterward, we compared the ratio of the AUC values between the years to illustrate prosperity differences that are related to the environmental conditions of the individual year.

## Results and discussion

### Environmental conditions

**Figure 2:**
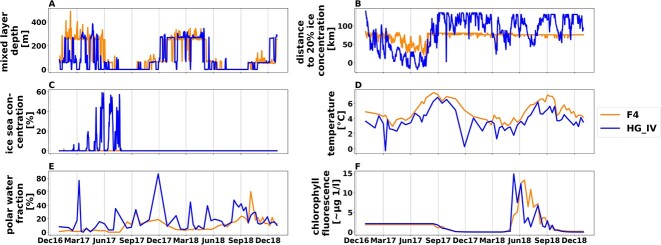
**Environmental data for the F4 (dark orange) and HG-IV (blue) location from 2017 to 2018**. The x-axis indicates the period from 01.01.2017 to 31.12.2018. The y-axis indicates: **A:** Mixed layer depth (Minimum of the estimated MLD) [m] **B:** Distance to 20% ice concentration (^*^) [m] **C:** Sea ice concentration [%] **D:** Temperature [°C] **E:** Polar water fraction [%] **F:** Chlorophyll a concentration (^*^^*^) [μL–1] ^*^Negative values indicate that the ice edge is south east of the mooring points at the blue curve March 2017 to September 2017) ^*^^*^Sensor did not work before August 2017.

A pronounced extension of the ice edge/MIZ into the WSC during the first half of 2017, compared to 2018, led to different environmental conditions in this part of the eastern Fram Strait. That MLR was similar to that expected for a seasonally ice-free Arctic Ocean, impacted by Atlantification. More specifically, eastern Fram Strait experienced extended sea ice melt during spring and early summer 2017. According to van Appen et al. 2021 [32], there were significant differences in environmental conditions between 2017 and 2018, with station HG-IV exhibiting more pronounced differences compared to the pure Atlantic Water station F4. This is best reflected by variability in the fraction of Polar Water, distance to the ice edge, ice concentration, and water column stratification (Fig. 2).

At HG-IV, the mixed layer depth was overall shallower from January to May 2017 compared to 2018 and F4 due to higher ice concentrations. Moreover, HG-IV was frequently impacted by the intrusion of Polar Water (PW) throughout the annual cycle, which is common for this region. Higher fractions of PW were observed for the period’s March, July to August, and November–December of 2017 compared to 2018, according to the RAS data. The intrusion of PW led to lower water temperatures. At HG-IV, temperatures were lower in spring 2017 compared to 2018—ice distances, defined as the distance to 20% ice coverage. At HG-IV, the distance to the ice edge was shorter in 2017 than in 2018 until August but was similar during the remaining months (Figure 2). From mid-August to November; water temperatures were higher in 2017 compared to 2018. In 2017, there was higher ice cover in Fram Strait and subsequent ice melt, resulting in a highly stratified melt water regime (MWR). In contrast, in 2018, an unstratified mixed layer dominated regime (MLR) was present [32].

At F4, ice distances were not significantly different between the two years. However, water temperatures were higher in 2017 compared to 2018 from mid-August to November. In this investigation, Station F4 serves as a reference for typical Atlantic environmental conditions for both years.

In the following section, we examined the behavior of eukaryotic microbes under distinct water regimes, namely meltwater and mixed layer conditions. To achieve this, we employed a top-down structure to delineate the temporal abundance changes for: (i) all ASVs, (ii) specific ASV clusters, and (iii) individual representative species.

### Preference of eukaryotic microbes for the different water regimes

There is a remarkable similarity in species composition between the two stations. A total of 50% (583) of all ASVs under inspection were detected at both stations, which we refer to as the core community. In contrast, 22% were unique to F4 (254 ASVs) and 28% to HG-IV (320 ASVs) (Figs S5 and S2). To determine the preferred water regime for microbial eukaryote taxa, we calculated the total relative abundance of each ASV per year and compared them between both years. This comparison was only possible at station HG-IV due to the differing conditions in both years. To achieve this, we sorted the ASVs into three groups based on the preferred water regime: the unstratified MLR, the highly stratified MWR, and an unspecified group. The MLR group comprises all temperate taxa, which were twice as abundant in HG-IV-2018 compared to HG-IV-2017 (n = 67 [11.49% of the core community]). In contrast, ASVs that were twice as abundant in HG-IV-2017 compared to HG-IV-2018 belong to the MWR group (n = 94 [16.12% of the core community]), which are referred to as polar taxa. The remaining ASVs were classified as an unspecified group (n = 422 [72.38% of the core community]). In the following steps, we focused on species that are sensitive to one of the water regimes that occurred. Notably, we identified 161 species in this study that showed a preference for a specific regime. These species were distributed between the MLR group (41.62%) and the MWR group (58.38%) (Table S5]).

### Cross spatio-temporal comparison

**Figure 3:**
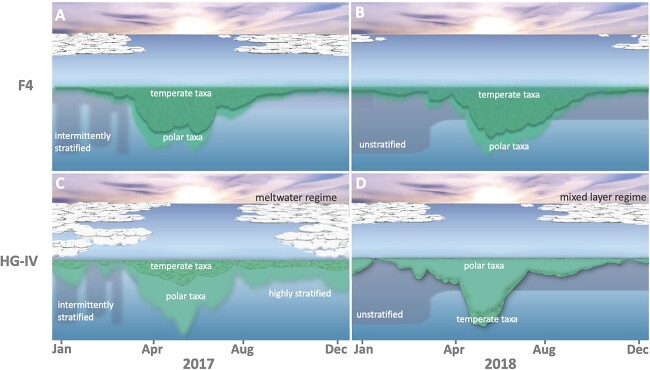
Effects of meltwater and mixed layer conditions on temperate (dark green) and polar (light green) taxa. The x-axis shows the months January through December from 2017 through 2018. The green areas reflect the relative abundances of temperate (dark green) and polar (light green) taxa. Since the data is relative, no quantification is given on the y-axis. The relative abundance curves of A and B were derived from water column samples from cluster F-06, and C and D from cluster H-06. **A:** Polar and temperate taxa are observed in similar abundances in the highly stratified meltwater regime at F4 in 2017. **B:** Similar abundances for polar and temperate taxa in the mixed layer regime at F4 in 2018. **C:** Reduced abundance of temperate taxa in the meltwater regime with high stratification at HG-IV in 2017. **D:** Reduced abundance of polar taxa in the mixed layer regime at HG-IV in 2018.

We compared both groups (MLR & MWR) to identify differences attributed to either location, HG-IV vs. F4 (Fig. 1), or the varying conditions between 2017 and 2018 (Fig. 3). To do so, we conducted two types of comparisons: (i) within each year, we compared the stations to each other and (ii) within each station, we compared the data from 2017 and 2018. First, we compared the relative abundance differences in 2017 between stations. We calculated the median of the MLR group and MWR group, respectively, and compared them. Our results showed that the median differences between the locations (Fig. 3A-D) of species favouring mixed-layer were 1.54 times larger than the median differences of the species favouring meltwater in 2017 (Table S5; see methods formula (2,3)). Furthermore, we confirmed this observation regarding the different medians by comparing the relative abundances of each ASV member in the aforementioned groups (one-sided Kolmogorov–Smirnov test *P*-value: 3.13E-05). In the next step, we repeated the same analysis for the year 2018.

In contrast to 2017, the median differences in 2018 of the meltwater-favouring species were 2.78 times greater than the median differences of the mixed-layer favouring species (Table S5; see methods formula (2,3)). Also, in this case, comparing the relative abundance of the particular ASVs could support this observation (one-sided Kolmogorov–Smirnov test *P*-value: 1.376E-14). Once we had distinguished dissimilarities among the stations, our attention turned to describing dissimilarities over the years (Fig. 3 A-D). This was motivated by the different water regimes observed in 2017 and 2018 [32]. Consequently, this examination enabled us to demonstrate how species abundance is influenced by varying environmental circumstances. Therefore we compared the relative abundance ratio of each group (MLR, MWR) between years (2017 vs. 2018). The difference between the two years (2017 and 2018) for each group was less significant at station F4 (MWR = 1.23 and MLR = 0.60), whereas at HG-IV, the discrepancy was approximately four times higher than that observed at F4 for the same years (MWR = 2.13 and MLR = 0.27), see Table S5. As a result for the following analysis, we used station F4 as a reference for constant environment because it is less influenced by meltwater conditions. In contrast, the HG-IV location offers the opportunity to study the effects of Atlantification in a seasonally ice-covered Arctic Ocean, conditions that are expected for the CAO in the near future [56]. For that, we examined how each other’s water regimes affected the relative abundance of the respective ASV. We aimed to determine whether polar or temperate ASVs were more resilient to the opposing condition. For the analysis, we specifically selected ASVs that are known to grow in polar or temperate conditions [57-64].

### Seasonal succession of eukaryotic microbes

**Figure 4:**
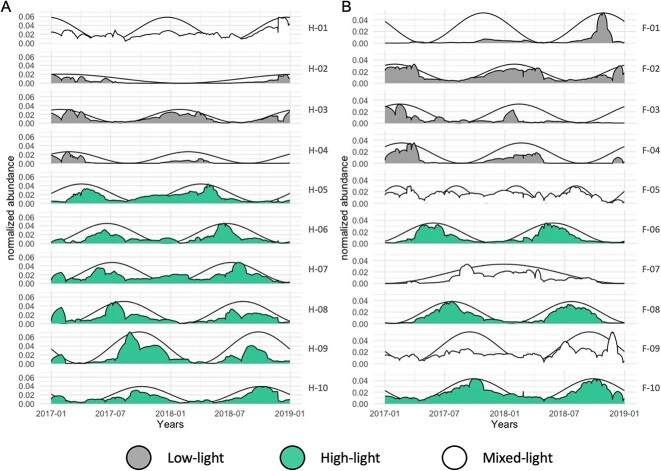
**Time-Series Clustering for both moorings spanning the years 2017-2018.** The x-axis indicates the period from 01.01.2017 to 31.12.2018. Black sinusoidal curves show the predicted seasonality of the entire cluster based on the dominant Fourier component. The respective relative abundance is shown for each cluster on the left yaxis. Cluster names are shown on the right. The clusters are sorted by phase which illustrates the time of maximal abundance of each community. Clusters are colored according to the three classes HL (green), LL (grey), and NA (white) introduced in the text. **A:** HG-IV, **B:** F4.

**Figure 5:**
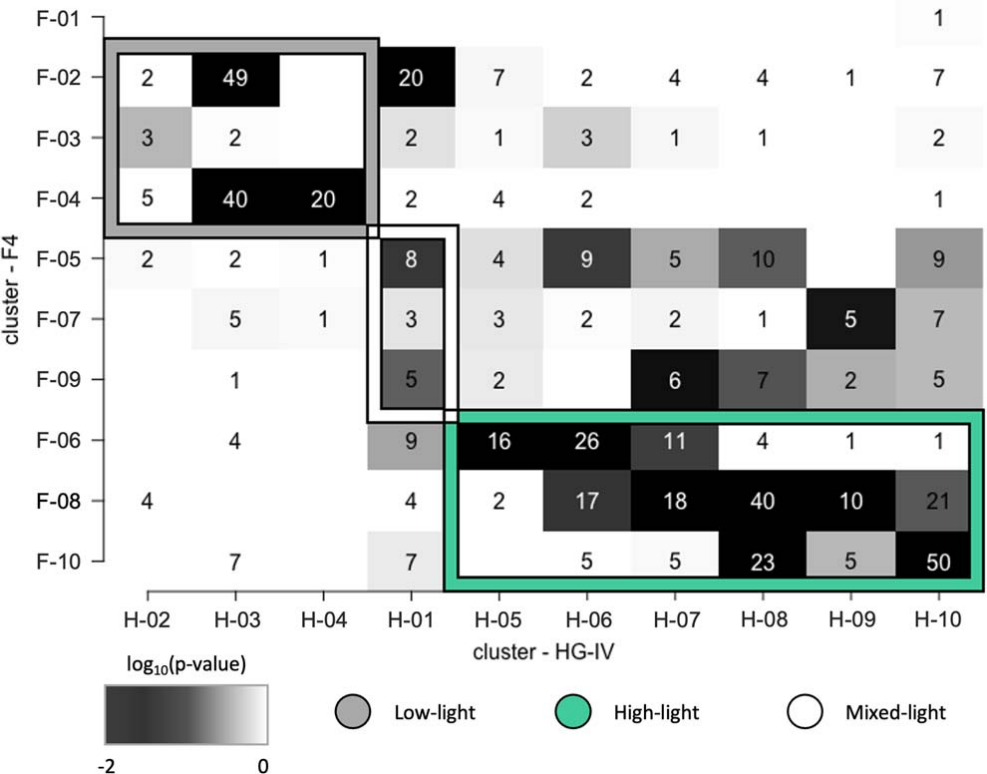
**Cluster overlap between F4 and HG-IV locations.** The clusters of F4 are plotted on the y-axis against the clusters of HG-IV. The numbers inside the boxes indicate how many ASVs are shared between two clusters The clusters of each location are sorted according to their classes: low-light (grey box frame), mix-light (white box frame) and high-light (green box frame) from top to bottom (F4) and from left to right (HG-IV). The background color of the boxes shows the significance of the overlap from dark (highly significant) to white (non significant).

To understand the seasonal succession of eukaryotic microbes, we analysed the phases obtained from the sinusoidal function after Fourier transformation. This allows us to determine the chronological timeline of the species in this region. Ten clusters of seasonally synchronized and ordered occurrences of eukaryotic microbial species were identified through community detection analysis of time-series data from the F4 and HG-IV moorings, which included 837 and 903 ASVs, respectively (Fig. 4, Table 1). The frequency obtained from the sinusoidal function (light grey) shows the number of high abundance periods of each community per year. Most clusters (85%) had two maxima, indicating that most organisms exhibit a seasonal occurrence with the highest abundance once a year (Fig. 4, Table 1). We divided the clusters based on their high abundance period into two classes of light conditions. The low-light (LL 0–2 hours sunlight per day) clusters include species with a high abundance phase in the low-light period from October to March when water temperature and distance to the ice edge are low. The high-light class (HL 2–24 hours sunlight per day) includes clusters, in which the high abundance phases coincide with the high-light period from March to October. All other clusters are collected in the mixed light (NA) class. This distinction allowed us to test the succession of the organisms regarding environmental factors per light condition separately. To investigate the commonalities and differences between the two moorings, we compared the species distribution in terms of abundance and seasonality. This analysis also enabled us to assess the succession and prosperity of common species in relation to the varying water regimes. In addition, we compared the time series cluster composition from HG-IV and F4 with each other to identify overlapping communities between both locations. For example, the similarity in cluster composition between the two moorings was highest during the high-light period, particularly between clusters H-06 and F-06 and clusters H-08 and F-08 (Fig. 5). The presence of these common ASVs at both mooring sites can be explained by a similar trend in the transportation of temperate organisms through the northward-flowing warmer Atlantic and the transportation of polar organisms through the intrusion of polar water from EGC. This pattern was also observed for zooplankton [65, 66]. On the other hand, the varying quantities of ASVs reaching each station because of variations in the influence of the two currents may also explain the biodiversity observed at these two locations (Fig. S1).

**Table 1:** Cluster overview with the 10 clusters for the moorings F4 and HG-IV.

Name	Type	#Peaks	cl_size	cl_size %	s-score	AUC17	AUC18	AUC17/18	AUC18/17	MS(abs)	MS(rel)
H-01	NA	2	72	9	0.18	6.9837	8.8812	0.7863	1.2717	12	16.67
H-02	LL	1	68	8	0.39	1.81	1.0084	1.7949	0.5571	52	76.47
H-03	LL	2	151	18	0.44	4.4387	4.1164	1.0783	0.9274	41	27.15
H-04	LL	2	50	6	0.83	1.7481	0.7188	2.432	0.4112	28	56
H-05	HL	2	76	9	0.33	5.2585	5.1262	1.0258	0.9748	37	48.68
H-06	HL	2	87	10	0.41	4.1827	4.7476	0.881	1.1351	21	24.14
H-07	HL	2	65	8	0.2	6.0155	6.5539	0.9179	1.0895	13	20
H-08	HL	2	113	14	0.32	6.6398	4.405	1.5073	0.6634	23	20.35
H-09	HL	2	33	4	0.53	7.9501	4.5641	1.7419	0.5741	9	27.27
H-10	HL	2	122	15	0.41	5.0174	5.0116	1.0012	0.9988	18	14.75
F-01	LL	2	27	3	0.66	0.6775	2.8243	0.2399	4.1687	26	96.3
F-02	LL	2	144	16	0.39	5.4081	5.1656	1.0469	0.9552	48	33.33
F-03	LL	2	33	4	0.46	3.289	1.228	2.6783	0.3734	18	54.55
F-04	LL	2	168	19	0.75	2.9621	1.8344	1.6148	0.6193	94	55.95
F-05	NA	1	61	7	0.06	6.194	5.2571	1.1782	0.8487	11	18.03
F-06	HL	2	109	12	0.58	3.9653	4.1903	0.9463	1.0567	37	33.94
F-07	NA	4	53	6	0.13	3.4143	3.529	0.9675	1.0336	24	45.28
F-08	HL	2	142	16	0.58	4.8684	4.4017	1.106	0.9041	26	18.31
F-09	NA	2	36	4	0.17	5.6129	7.6206	0.7365	1.3577	8	22.22
F-10	HL	2	130	12	0.34	7.0415	7.0604	0.9973	1.0027	28	21.54

The cluster names, light types (high-light (HL), low-light (LL), mixed-light (NA)), the number of peaks and the total cluster size of ASV and the percent size, the s-score that measures the seasonality, the area under the curve (AUC) for both years (see methods), the quotients of those years and the number of ASV that only occur on this mooring: absolute (MS(abs)) and relative values (in %) (MS(rel)) (MS: mooring specific).

### Low-light period

During the low-light period from October to March, four distinct clusters (F-01, F-02, F-03, F-04 at F4; H-02, H-03, and H-04 at HG-IV) exhibited an ordered appearance, collectively representing around 40% of the total ASVs and 50% of the total reads at both stations. The clusters were dominated by heterotrophic Dinoflagellates, parasitic Syndiniales, and other small heterotrophic flagellates like MAST and Picozoa (Fig. S4). This composition aligns with previous reports of microbial diversity during the low-light period in the Arctic Ocean [66-68], possibly linked to feeding on bacteria [67]. Notably, diatom ASVs were present in all low-light clusters, exhibiting substantial relative abundances, with higher proportions at HG-IV compared to F4 (Fig. S4). These diatoms, including ice-associated genera such as *Melosira arctica*, *Navicuales sp.,* or *Attheya sepentrionalis* (Fig. S4Table S4), are adapted to low light and colder temperatures [69] or residing under the ice [70]. The source of these diatoms in the water column during winter at HG-IV is attributed to physical exchange processes at the water-sea ice interface and advection. The persistence of diatoms, particularly Bacillariophyceae, during the polar night in ice-covered waters has been observed previously [67] and their survival strategies, possibly involving resting stages like spores or cysts [71]), influence the composition of Arctic phytoplankton during early spring. Thistaxon-specific survival contributes to diatoms gaining a competitive advantage in the Arctic phytoplankton community when sunlight returns, facilitated by their chlorophyll storage throughout the polar night [72].

### High-light period

**Figure 6:**
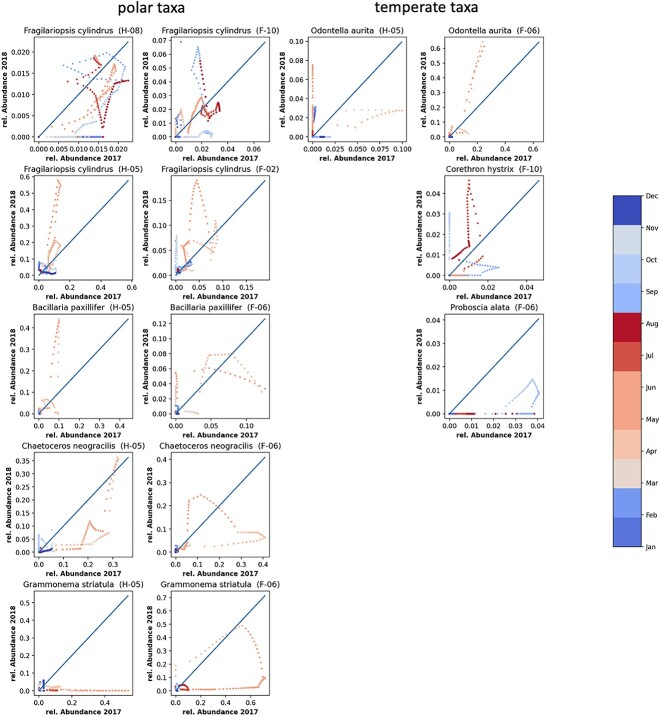
**Correlation between the relative abundances of selected ASVs in 2017 vs 2018**: The diagonal (blue line) indicates the line on which abundances in 2017 and 2018 would be identical. On the left side (first and second columns) selected polar taxa are displayed, where the first column shows the species at HG-IV and the second column the same ASV at F4. The right side shows selected temperate taxa, where the third column displays species at HG-IV and the fourth column the same ASV at F4. The dots indicate: *Fragilariopsis cylindrus* (ASV207: H-08, F-10), *Fragilariopsis cylindrus* (ASV16: H-05, F-02), *Bacillaria paxillifer* (ASV98: H-03, F-06), Chaetoceros neograciis (ASV17: H-05, F-06), *Grammonema stratula* (ASV33: H-05, F-06), Odontella aurita (ASV96:H-05, F-06), Corethron hystrix (ASV172: F-10), *Proboscia alata* (ASV947: F-06), Color bar and colored dots indicate month of the year from blue (winter) to red (summer).

The high-light period (March to October), distinct clusters (F-06, F-08, F-10 at F4;H-05, H-06, H-07, H-08, H-09, H-10 at HG-IV) sequentially emerged, collectively constituting ~50% of mooring-specific ASVs (Table 1). The community composition of the earlier high-light clusters in 2017 at HG-IV resembled that of early high-light in 2018 at F4 (Fig. 5), suggesting a shared community initiation (Table S3, Table S4). Throughout this period, diatoms, alongside dinoflagellates and other autotrophic taxa, were prevalent (Fig. S4, Table S3). Diatom sequences exhibited a sequential appearance during spring, aligning with Arctic diatoms like *Fragilariopsis cylindrus, Bacillaria paxilifer, Chaetoceros neogracilis*, and *Grammonema striatula* [73-75] (Table S3) Their major contribution to the pelagic spring bloom emphasized the polar character of the spring bloom community at HG-IV [7, 27, 76]. Notably, *Grammononema striatula* and *C. neogracilis,* polar taxa, were abundant in the first high-light cluster (F-06) at. In contrast to the Arctic diatoms dominating the spring bloom at HG-IV, the temperate diatom *Odontella aurita* [77] ranked among the five most abundant diatoms in the early spring cluster at F4. This suggests the influence of Atlantic Water, transporting organisms from warmer, temperate waters (Fig. 6). *O. aurita*, a key contributor to spring blooms in the German Bight [78], further supports the idea that it thrives in warm, nutrient-rich waters.

Differences in diatoms community composition between F4 and HG-IV became more pronounced in the late summer clusters (H-08 and F-10), which peaked after July. Cluster H-08 at HG-IV was dominated by sea-ice-associated diatoms like *Melosira arctica* and related taxa [70], comprising 57% of the total diatom abundance in this cluster (Table S4 H-08). In contrast, the late cluster F-10 at F4 was dominated by *Pseudonitzschia sp.* representing 38% of the total diatom abundance (Table S4 F-10). The late summer cluster F-10 at F4 also contained significant quantities of *Corethron hystrix* and *Proboscia alata,* two diatoms thriving in temperate waters [79, 80] highlighting the influence of Atlantic Water on the diatom community at F4. Cluster H-09 at HG-IV, representing 28% of the total abundance of diatoms (Table S3), was dominated by the genus Pseudonitzschia, known for year-round blooms with peaks in late August or early September [81]. Studies have shown that this diatom undergoes blooming throughout the year, typically exhibiting a minor bloom in June, followed by a more substantial bloom in late August or early September [81]. Other major Arctic pelagic autotrophs, such as *Phaeocystis sp.*, *Chaetoceros socialis* and *Micromonas sp.,* were predominantly found in clusters with high light levels (Table S3). Specifically, *Phaeocystis pouchetii* was most abundant in early spring clusters (H-05 and F-06) accounting for 16% and 11% of the total abundance, respectively (Table S3 H-05, F-06), consistent with findings from the Western Spitsbergen Current (WSC) and under the ice north of Svalbard [82, 83].

### Impact of sea-ice melt on seasonal phytoplankton dynamics and consequences for bloom phenology in Atlantic waters

**Figure 7:**
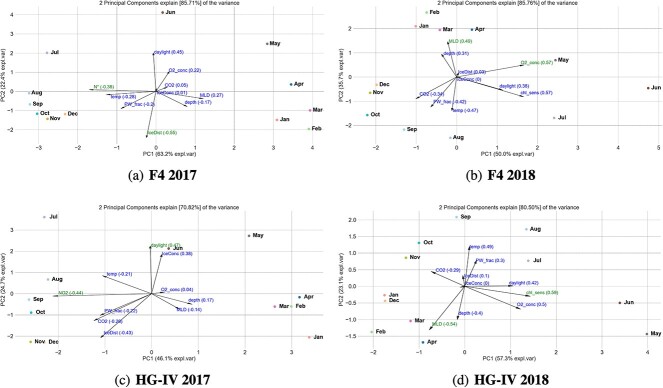
**Biplots for both mooring and both years:** First PCA component plotted versus the second PCA component of the abundance values for the ASV and the environment conditions on monthly bases aggregated. The arrows correspond to the most relevant features. The length of the arrow corresponds to the feature importance associated with this arrow. **A:** HG-IV 2017, **B:** HG-IV2018, **C:** F4 2017 and **D:** F4 2018.

The different environmental conditions observed in 2017 and 2018 did not seem to affect the order of the annually recurring community clusters at F4 and HG-IV. Instead, changes in environmental conditions resulted in differences in their persistence, abundance amplitude, and integrated abundances (Fig. 4 A and B). At F4, environmental conditions during the high light periods of 2017 and 2018 were similar. In consequence, the integrated seasonal cluster abundance, reflected by the area under the curve, did not significantly change from one year to the other (Table 1). In contrast, we observed differences between both years for HL and LL periods at HG-IV (Fig. 7). According to our data, the changes in environmental conditions, associated with sea-ice melt in spring and summer 2017 at HG-IV, might have significantly affected the communities during high-light periods. For example, these changes can be observed in the high-light cluster H-09 (Table 1). The last period of the cluster (2018) shows a 1.7-fold decrease in abundance compared to the first period (2017). Despite the area under the curve of the early high-light clusters (H-05, H-06, and H-07) showing almost no difference between the two years at HG-IV, the amplitude was much lower in 2017 compared to 2018 (Table S2). This observation suggests that the growth rates in 2017 were lower. It is important to note that the organism abundances only reflect relative proportions of the filtered samples. However, in 2017 the RAS was below the productive layer for at least the first half of the high-light period [35], which may explain the lower relative abundances.

Polar pelagic taxa, such as *C. neogracilis* and *Grammonema striatula*, were dominant (compared to other Bacillariophyta) in the first clusters of the high-light period at both stations (H-05, F-06, Table S3). These species are more robust to variation in ice coverage. In contrast, the contribution of *Fragillariopsis cylindrus* to the spring cluster H-05 was greater at HG-IV than at F-06, as indicated in Table S3. During the spring of 2017 at HG-IV, lower relative abundances of *F. cylindrus* may suggest lower growth rates, which could be attributed to higher ice coverage at this station. *F. cylindrus* and *Bacillaria pacillifer* were among the ASVs with the ten highest relative abundances at both stations. They had higher relative abundances during the spring at HG-IV compared to F4 in the observation period, as shown in Table S4 and Fig. 6. This was likely because they benefited from lower ice concentrations and comparatively higher water temperatures at HG-IV during the spring of 2018 compared to 2017 (Fig. 6). This observation suggests that these polar taxa are not strictly dependent on polar conditions and can tolerate or benefit from Atlantic influence.

*O. aurita*, a temperate taxon occurring at both stations, benefits at both stations from warmer temperatures. The contribution of this temperate species in cluster H-05 was negligible, accounting for only 1% of total abundance, with a further decrease in 2017 to 0.81%, indicating that it struggles to thrive under the ice. In contrast, at mooring F4, its contribution to the spring cluster F-06 was high in both years (Table S3) as temperatures were in a similar range. During the later part of the season in HG-IV, the area under the curve of cluster H-08 showed a 1.5-fold increase in 2017 compared to 2018, as indicated in Table 1. This cluster mainly comprised typical sea-ice-associated diatoms like *M. arctica*, *Fragillariopsis sublineata* and -*cylindrus*, and *Chaetoceros rostratus*. Interestingly, these diatoms did not contribute significantly to the phytoplankton community at F4 during the same time of the year. This indicates a sea-ice melt-related release of sea-ice-associated taxa. The environmental conditions existing at this time, especially meltwater stratification, promoted their bloom in the Atlantic Water of Fram Strait (Tables S3 and S4).

During the specified time frame, there was a notable decrease in the prevalence of polar spring phytoplankton species at the start of the season, accompanied by a corresponding increase in the abundance of ice-associated phytoplankton species during the autumn of 2017. It is worth noting that the peak abundance of ice-associated phytoplankton species usually occurs later in the season in the CAO [84-86]. Ice-associated phytoplankton is less present at HG-IV in 2018 (ice-free year) and does not significantly contribute to the autumn community at ice-free station F4 in either year.

## Conclusion

In this study, we compared the dynamics of phytoplankton ASVs from two locations in the Fram Strait (moorings HG-IV and F4) as recorded in 2017 and 2018 (Fig. 3). Although data from only 2 years are not necessarily representative of the long-term development of environmental parameters, these particular years exhibit conditions that make them appear ideal for comparing current conditions with those expected in the future in an Atlantified CAO. This comparison supports a new perspective on how the eukaryotic microbial community in the Central Arctic Ocean might change in the near future. Climate change will likely lead to an ice-free Central Arctic Ocean in summer but ice-covered in winter, as suggested by some climate model scenarios [13].

In our analysis, we could show that a MWR can strongly influence arctic micro-eukaryotes on several levels and that phytoplankton bloom phenology in 2017 is a result of an increased sea ice melt [32]. We could extend previous observations about the influence of sea-ice melt on community dynamics and carbon export. We propose that sea ice melt and the resulting environmental conditions are putative key drivers of microbial eukaryotic community composition and bloom phenomenology. Our observations suggest that polar pelagic and ice-associated taxa (such as *F. cylindrus* or *M. arctica*) are relatively tolerant of more Atlantic oceanographic conditions. In contrast, temperate taxa (such as *O. aurita* or *P. alata*) have limited potential to persist in colder ice-impacted waters. Thus, we hypothesize that sea-ice melt in the MIZ may hinder the northward expansion of temperate Atlantic taxa towards the CAO. This trend will continue even as Atlantic oceanographic conditions move further northwards.

## Supplementary Material

Supplementary_information_ycae027

FigS1_ycae027

FigS2_ycae027

FigS3_ycae027

FigS4_ycae027

FigS5_ycae027

TableS1_ycae027

TableS2_ycae027

TableS3_ycae027

TableS4_ycae027

TableS5_ycae027

## Data Availability

Raw data can be obtained from the European Nucleotide Archive (ENA) at https://www.ebi.ac.uk/ena/browser/view/PRJEB43905. The datasets generated during and/or analysed during the current study are available in the gitlab repository, https://gitlab.com/qtb-hhu/qtb-sda/framstrait_1718.
