# Supplementary material for: Sea-ice melt determines seasonal phytoplankton dynamics and delimits the habitat of temperate Atlantic taxa as the Arctic Ocean atlantifies
Source: ISME Commun. 2024 Feb 27;4(1):ycae027. doi: 10.1093/ismeco/ycae027 (PMC10955684; doi:10.1093/ismeco/ycae027)
Supplement: Supplementary_information_ycae027 [file supplementary_information_ycae027.pdf]

Supplementary information

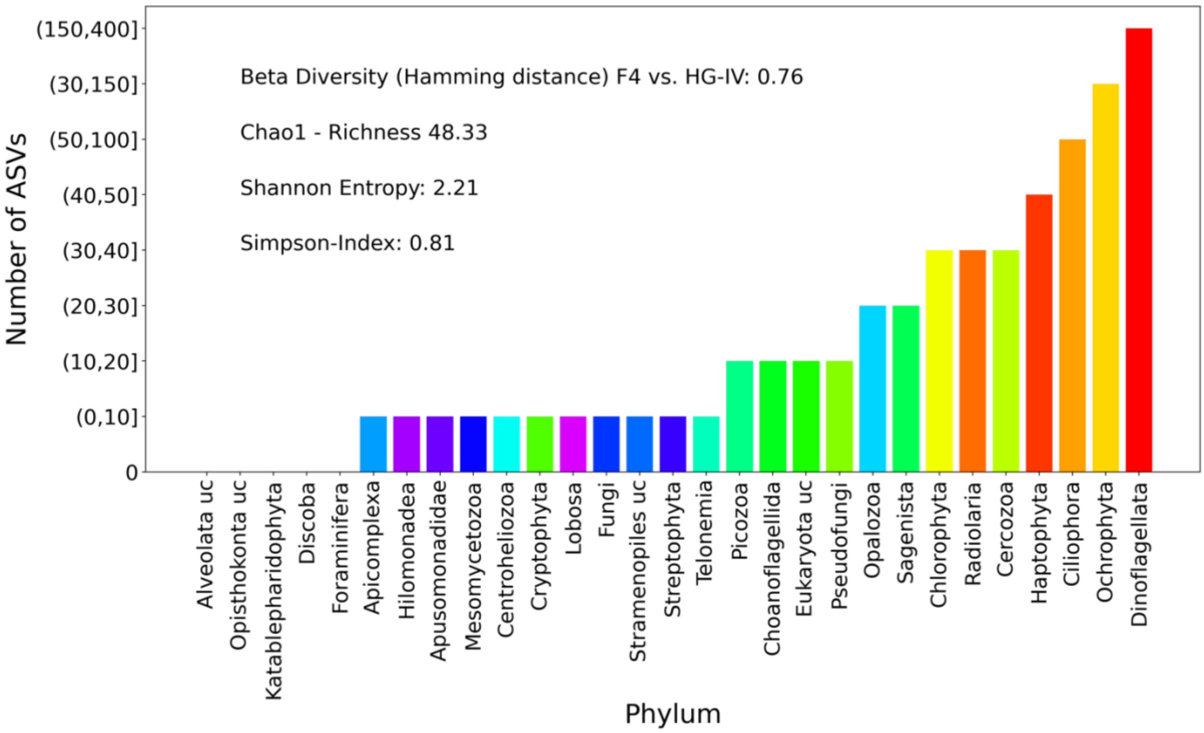

Figure S1: **Bio Diversity on Phylum Level:** The Bar plot show the number of ASV of the corresponding Phylum. Beta Diversity (Hamming distance) between both moorings and the alpha diversity for this mooring are displayed. The Richness by Chao1, the Shannon Entropy and the Simpson-Index as measurements for the alpha diversity are also calculated. **A**: HG-IV, **B**: F4.

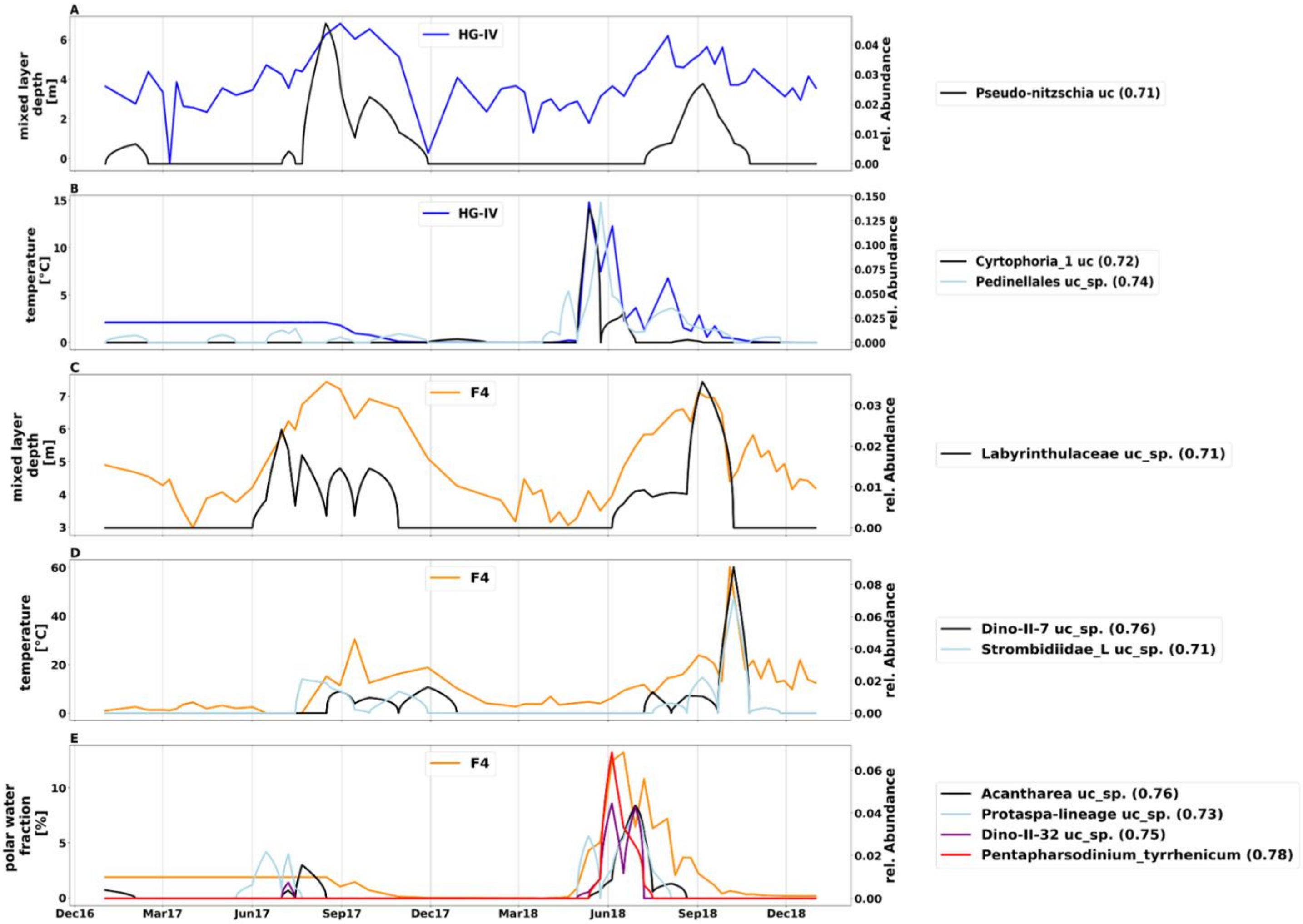

Figure S2: **Correlation between environment conditions and Species (ASVs):** Species with coefficient greater then 0.7, the value bracket indicate Pearson correlation coefficient are shown. **A**: HG-IV: mixed-layer depth, **B**: HG-IV: temperature, **C**: F4: mixed-layer depth, **D**: F4: temperature, **E**: F4: polar water fraction.

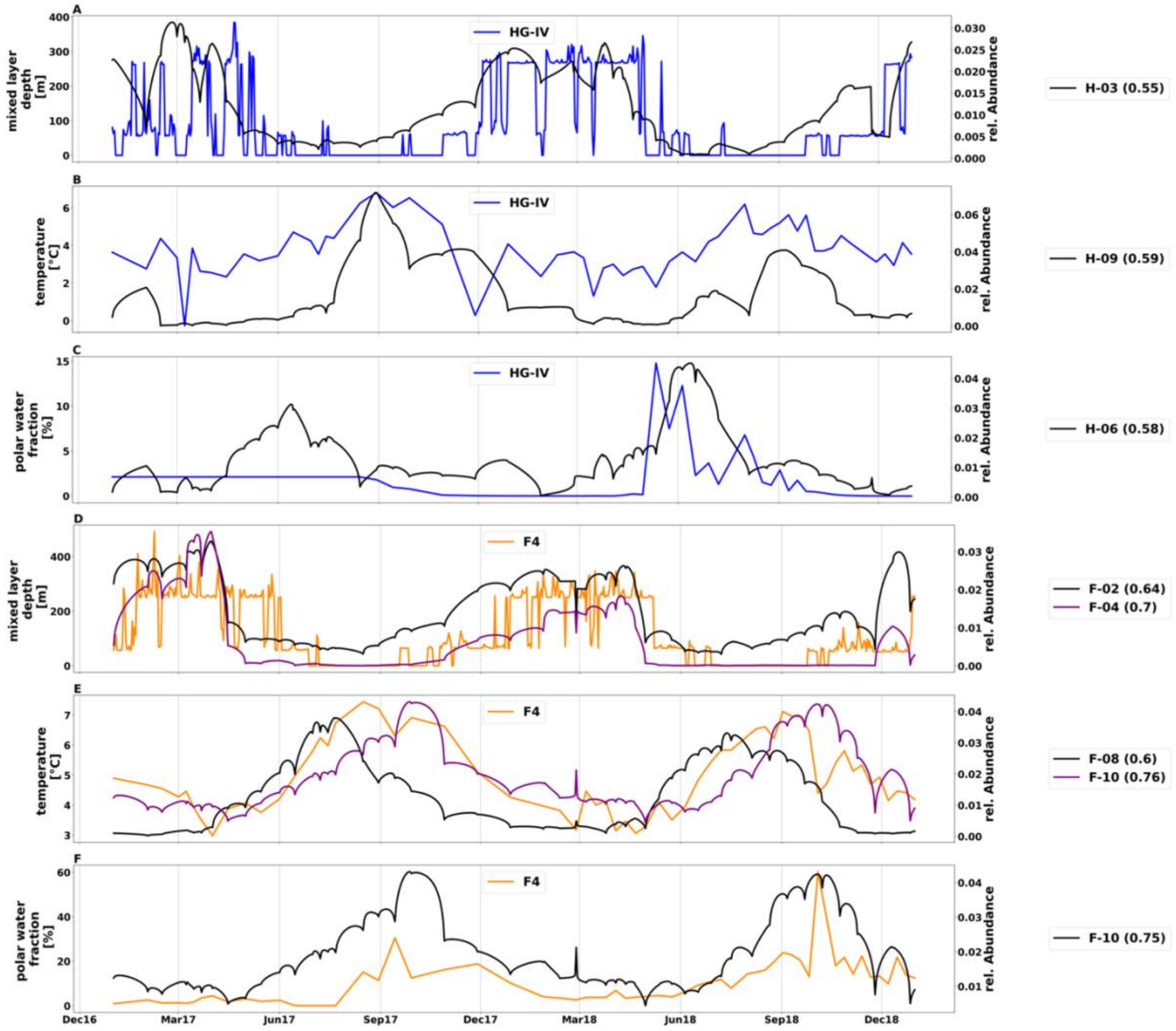

Figure S3: **Correlation between environment conditions and clusters:** Clusters with coefficient greater then 0.55, the value bracket indicate Pearson correlation coefficient are shown. **A**: HG-IV: mixed-layer depth, **B**: HG-IV: temperature, **C**: HG-IV: polar water fraction, **D**: F4: mixed-layer depth, **E**: F4: temperature, **F**: F4: polar water fraction.

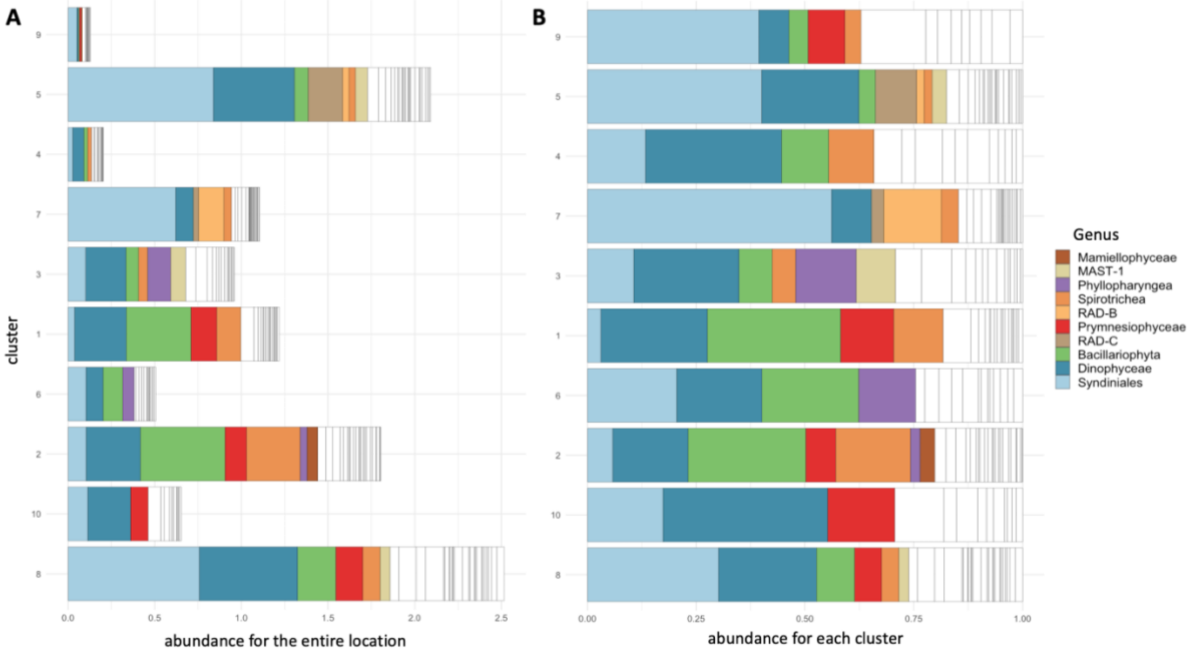

(a) **F4**

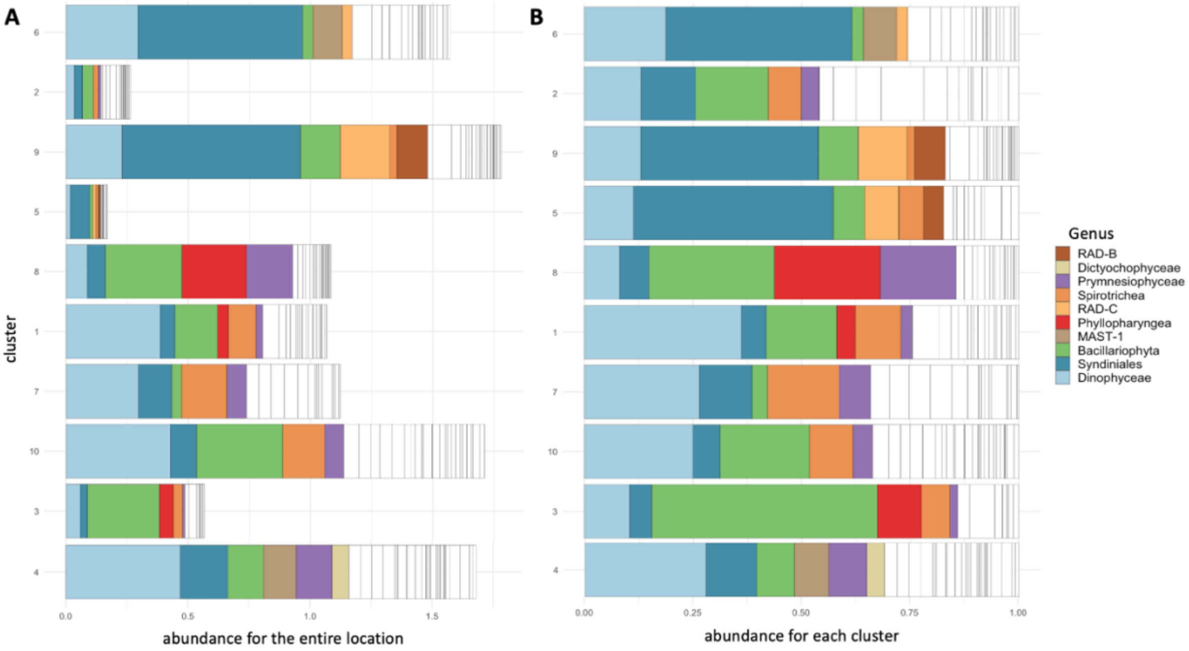

(b) **HG-IV**

Figure S4: **Taxa bar-plot showing the top then microbial communities for both moorings.** Clusters are grouped according to their seasonality on the y-axis. Relative sequence abundances (%) of the ten most abundant eukaryotes classes, with all other genera grouped in "Other " (colored in white) are shown. The top 10 genera for HG-IV are shown ascending, starting with Dinophyceae (skyblue), Syndiniales (blue), Bacillariophyta (light green), MAST-1 (light brown), Phyllopharyngea (red), RAD-C (light orange), Spirotichea (orange), Prymnesiophyceae (purple), Dictyochophyceae (beach) and RAD-B (brown). The top 10 genera for F4 are shown ascending, starting with Syndiniales (skyblue), Dinophyceae (blue), Bacillariophyta (light green),

RAD-C (light brown), Prymnesiophycaeae (red), RAD-B (light orange), Spirotichea (orange), Phyllopharyngea (purple), MAST-1 (beach) and Mamiellophyceae (brown). The x-axis indicates **A**: portion on the whole mooring for HG-IV, **B**: portion on the certain cluster for HG-IV., **C**: portion on the whole mooring for F4 and **D**: portion on the certain cluster for F4.

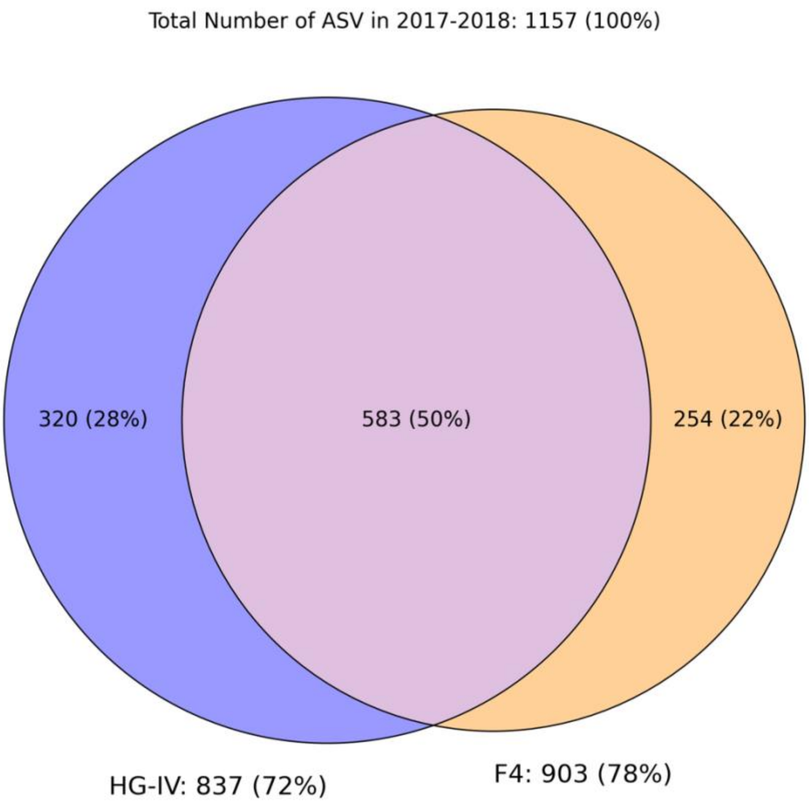

Figure S5: **Different ASV Communities for F4 and HG-IV:** Venn Diagram showing the Union (combination of cyan, dark orange and green), the Intersection (green) and the Set-Differences (F4 dark orange, HG-IV cyan) of the ASV for the F4 and HG-IV locations for 2017-2018.

Table S1: **Selected Species:** The species, the asv-name, the area under the curve 2017 (AUC17) and area under the curve 2018 (AUC18) and the quotients are shown.

**Species**	**asv**	**Cluster**	**AUC17**	**AUC18**	**Quotient 17/18**	**Quotient 18/17**
Odontella_aurita	euk_asv96	H-05	3,2281	4.5659	0.707	1.4144
Fragilariopsis_cylindrus	euk_asv207	F-10	4.6039	4.3962	1.0472	0.9549
Fragilariopsis_sublineata	euk_asv35	F-08	23.7127	14.1505	1.6757	0.5967
Fragilariopsis_sublineata	euk_asv35	H-08	25.9609	13.7689	1.8855	0.5304
Pseudo-nitzschia_sp.	euk_asv9	F-10	31.291	30.224	1.0353	0.9659
Pseudo-nitzschia_sp.	euk_asv9	H-09	66.0964	42.8685	1.5418	0.6486
Melosira_arctica	euk_asv244	H-08	2.2228	0.247	8.9992	0.1111
Melosira_arctica	euk_asv632	H-02	5.2386	0	∞	0

**cluster**	**max_peak_date_17**	**max_peak_17**	**max_peak_date_18**	**max_peak_18**
H-01	20.02.17	0.03	26.11.18	0.059
H-02	02.01.17	0.02	17.12.18	0.019
H-03	25.02.17	0.031	31.12.18	0.027
H-04	18.02.17	0.026	01.04.18	0.011
H-05	20.04.17	0.033	25.04.18	0.044
H-06	13.06.17	0.031	12.06.18	0.045
H-07	26.05.17	0.036	29.07.18	0.048
H-08	17.07.17	0.05	07.07.18	0.033
H-09	29.08.17	0.072	07.09.18	0.041
H-10	17.09.17	0.031	18.10.18	0.039
F-01	29.10.17	0.007	27.10.18	0.052
F-02	31.03.17	0.033	16.12.18	0.03
F-03	12.02.17	0.033	26.01.18	0.023

F-04	31.03.17	0.035	08.04.18	0.018
F-05	19.03.17	0.03	29.07.18	0.029
F-06	11.06.17	0.032	07.05.18	0.035
F-07	09.09.17	0.033	12.04.18	0.024
F-08	21.07.17	0.038	13.07.18	0.033
F-09	10.09.17	0.026	24.11.18	0.055
F-10	27.09.17	0.043	03.10.18	0.042

Table S2: **F4 and HG-IV Cluster max peaks per year:** For each mooring, for cluster and each year, the maximal peak date and the maximal peak are shown.

Table S3: **F4 and HG-IV Top Abundance species by sum off all ASV of this species:** For each cluster the top 10 species with their relative abundance and the portion of relative abundance of the cluster, calculated by summing up the ASVs relative abundance values for each of the both year are shown. The species that are referred in the text are colored light-gray.

	H-01 (NA)	H-02 (LL)	H-03 (LL)	H-04 (LL)	H-05 (HL)
1	Dino-I-1 uc_sp. (328.25) (28.61)	Labyrinthula_sp. (14.21) (7.38)	RAD-C uc_sp. (146.17) (11.25)	Dino-II-7 uc_sp. (15.44) (12.5)	Ephelota_mammillata (192.8) (24.37)
2	Dinophyceae uc (94.08) (8.2)	Chrysophyceae C uc_sp. (9.77) (5.08)	Dino-II-10-and-11 uc_sp. (111.53) (8.58)	RAD-C uc_sp. (9.71) (7.86)	Phaeocystis_pouchetii (127.1) (16.07)
3	Picozoa uc_sp. (59.76) (5.21)	Chaetoceros_sp. (7.68) (3.99)	Dino-I-4 uc_sp. (100.16) (7.71)	Dinophyceae uc (8.62) (6.97)	Fragilariopsis_cylindrus (45.02) (5.69)
4	MAST-1A uc_sp. (41.14) (3.59)	Allium_fistulosum (7.14) (3.71)	Dino-II-7 uc_sp. (73.08) (5.62)	Dino-II-20 uc_sp. (6.91) (5.59)	Gyrodinium_helveticum (41.04) (5.19)
5	Dino-I-4 uc_sp. (34.8) (3.03)	Pelagostrobilidium_sp. (7.11) (3.7)	RAD-B-Group-IV uc_sp. (49.36) (3.8)	Dino-I-5 uc_sp. (4.89) (3.96)	Grammonema_striatula (33.23) (4.2)
6	MAST-1B uc_sp. (32.1) (2.8)	Blastodinium_mangini (6.36) (3.3)	MAST-3F uc_sp. (46.3) (3.56)	Dino-II-6 uc_sp. (4.66) (3.77)	Chaetoceros_neogracilis (32.89) (4.16)
7	Chaetoceros_socialis (31.04) (2.7)	Pleurosigma_intermedium (5.5) (2.86)	Prorocentrum_sp. (45.74) (3.52)	Dino-I-4 uc_sp. (4.55) (3.68)	Dino-I-1 uc_sp. (26.65) (3.37)
8	Stephanoecidae D uc_sp. (29.77) (2.6)	Melosira_arctica (5.24) (2.72)	Dino-I-3 uc_sp. (37.98) (2.92)	Salpingacantha_sp. (3.57) (2.89)	Bacillaria_paxillifer (18.6) (2.35)
9	RAD-C uc_sp. (28.74) (2.5)	Challengeriidae uc_sp. (5.16) (2.68)	Polarella_glacialis (31.96) (2.46)	Dino-I-2 uc_sp. (3.55) (2.87)	Eukaryota uc (15.51) (1.96)
10	Dino-II-7 uc_sp. (28.57) (2.49)	Cryothecomonas-lineage uc_sp. (4.73) (2.46)	Dino-II-6 uc_sp. (31.68) (2.44)	Dino-II-3 uc_sp. (3.25) (2.63)	Pyramimonadales uc_sp. (14.35) (1.81)
	H-06 (HL)	H-07 (HL)	H-08 (HL)	H-09 (HL)	H-10 (HL)
1	Gyrodinium_fusiforme (78.28) (10.06)	Gyrodinium_helveticum (50.81) (6.21)	Thalassiosira uc (90.48) (7.24)	Pseudo-nitzschia_sp. (114.88) (27.78)	Dinophyceae uc (122.14) (9.97)
2	Dinophyceae uc (63.8) (8.2)	Leegaardiella_sp. (47.58) (5.81)	Peridiniales uc (87.34) (6.99)	Raphid-pennate uc (44.32) (10.72)	MAST-1C uc_sp. (65.21) (5.32)
3	Pseudo-nitzschia_sp. (61.75) (7.94)	Noelaerhabdaceae uc (40.49) (4.95)	Dinophyceae uc (65.32) (5.23)	Suctoria uc_sp. (41.64) (10.07)	Chrysochromulina_sp. (46.54) (3.8)
4	Gyrodinium_heterogrammum (60.11) (7.72)	Woloszynskia_halophila (39.36) (4.81)	Strombidiidae_H uc_sp. (47.53) (3.8)	Thalassiosira_sp. (23.49) (5.68)	Gymnodiniales uc (44.05) (3.59)
5	Leegaardiella_sp. (34.3) (4.41)	Strombidiidae_M uc_sp. (37.08) (4.53)	Picozoa uc_sp. (46.06) (3.69)	Pseudo-nitzschia uc (19.0) (4.6)	Picozoa uc_sp. (36.32) (2.96)
6	Chytriodinium_roseum (28.63) (3.68)	Pirsonia uc_sp. (35.92) (4.39)	Fragilariopsis_sublineata (39.73) (3.18)	Dino-II-10-and-11 uc_sp. (18.1) (4.38)	Peridiniales uc (34.96) (2.85)
7	Strombidium_M uc (23.57) (3.03)	Dino-I-1 uc_sp. (35.25) (4.31)	Aplanochytrium_sp. (34.83) (2.79)	Dadayiella_ganymedes (14.22) (3.44)	Fragilariopsis uc (33.77) (2.75)
8	Protaspa-lineage uc_sp. (17.92) (2.3)	Eukaryota uc (30.06) (3.67)	Chaetoceros_danicus (31.4) (2.51)	Gyrodinium_helveticum (12.42) (3.0)	MAST-1A uc_sp. (27.77) (2.27)
9	Prasinoderma_sp. (16.43) (2.11)	MOCH-2 uc_sp. (28.53) (3.49)	Gyrodinium_fusiforme (30.89) (2.47)	Dinophyceae uc (12.31) (2.98)	Dino-II-23 uc_sp. (25.87) (2.11)
10	Mediophyceae uc (15.93) (2.05)	Pelagococcus_sp. (28.15) (3.44)	Woloszynskia_sp. (30.46) (2.44)	MAST-3I uc_sp. (11.92) (2.88)	Pelagophyceae uc_sp. (25.46) (2.08)
	F-01 (LL)	F-02 (LL)	F-03 (LL)	F-04 (LL)	F-05 (NA)
1	Dino-I-1 uc_sp. (27.25) (28.78)	Dino-I-4 uc_sp. (151.52) (9.91)	Dinophyceae uc (23.09) (15.45)	Dino-II-7 uc_sp. (136.11) (16.85)	Ephelota_mammillata (97.48) (13.92)
2	Pelagophyceae uc_sp. (14.12) (14.91)	RAD-C uc_sp. (144.56) (9.46)	Gymnodiniaceae uc (9.75) (6.53)	Dino-II-10-and-11 uc_sp. (49.74) (6.16)	Dinophyceae uc (69.37) (9.91)
3	Chrysochromulina_sp. (5.97) (6.3)	Dino-I-1 uc_sp. (108.07) (7.07)	Cryothecomonas_aestivalis (9.62) (6.44)	RAD-B-Group-I uc_sp. (48.32) (5.98)	Peridiniales uc (44.84) (6.41)
4	Dinophyceae uc (5.16) (5.45)	Dinophyceae uc (104.94) (6.87)	Stephanoecidae D uc_sp. (9.38) (6.28)	Dinophyceae uc (42.55) (5.27)	Pirsonia uc_sp. (42.14) (6.02)
5	Chrysophyceae C uc_sp. (3.92) (4.14)	Dino-II-7 uc_sp. (63.2) (4.14)	Chaetoceros_sp. (8.17) (5.47)	RAD-B-Group-IV uc_sp. (40.5) (5.01)	MAST-1C uc_sp. (41.4) (5.91)
6	Tintinnopsis_07_sp. (3.49) (3.69)	Gyrodinium_helveticum (60.41) (3.95)	Dino-I-1 uc_sp. (7.71) (5.16)	Dino-I-5 uc_sp. (34.09) (4.22)	Stephanoecidae H uc_sp. (28.6) (4.08)
7	Dino-II-10-and-11 uc_sp. (3.03) (3.19)	Prorocentrum_sp. (56.14) (3.67)	Pterosperma_cristatum (7.58) (5.07)	Dino-I-2 uc_sp. (32.04) (3.97)	Heterocapsa_nei/rotundata (25.92) (3.7)
8	MAST-3I uc_sp. (2.94) (3.1)	Picozoa uc_sp. (45.32) (2.97)	Strombidiida uc (7.13) (4.77)	Dino-II-6 uc_sp. (27.08) (3.35)	Dinoflagellata uc (22.26) (3.18)
9	Dino-II-23 uc_sp. (2.72) (2.87)	Gymnodiniales uc (43.83) (2.87)	Gymnodinium_sp. (6.45) (4.32)	RAD-C uc_sp. (22.75) (2.82)	Chaetoceros_debilis_1 (21.76) (3.11)
10	Asterotremella uc (2.71) (2.87)	Dino-II-10-and-11 uc_sp. (38.23) (2.5)	Melosira_arctica (6.11) (4.09)	Dino-II-20 uc_sp. (22.4) (2.77)	MAST-1B uc_sp. (21.71) (3.1)
	F-06 (HL)	F-07 (NA)	F-08 (HL)	F-09 (NA)	F-10 (HL)
1	Phaeocystis_pouchetii (100.33) (11.28)	Dinophyceae uc (34.82) (9.43)	Thalassiosira uc (98.57) (7.48)	Dino-I-1 uc_sp. (58.84) (12.33)	Dino-I-1 uc_sp. (355.59) (19.36)
2	Gyrodinium_fusiforme (81.6) (9.17)	Thalassiosira_sp. (33.67) (9.12)	Gyrodinium_heterogrammum (57.51) (4.37)	Picozoa uc_sp. (53.99) (11.32)	Dino-II-10-and-11 uc_sp. (96.83) (5.27)
3	Grammonema_striatula (71.1) (7.99)	Raphid-pennate uc (33.19) (8.99)	Leegaardiella_sp. (53.82) (4.09)	Dinophyceae uc (50.97) (10.68)	Dinophyceae uc (88.33) (4.81)
4	Strombidium_M uc (40.76) (4.58)	Dino-II-10-and-11 uc_sp. (25.62) (6.94)	Strombidiidae_M uc_sp. (47.87) (3.63)	Noelaerhabdaceae uc (43.15) (9.04)	Aplanochytrium_sp. (71.99) (3.92)
5	Woloszynskia_halophila (32.18) (3.62)	Suctoria uc_sp. (20.84) (5.64)	Fragilariopsis uc (45.12) (3.43)	Gyrodinium uc (37.71) (7.9)	Peridiniales uc (69.32) (3.77)
6	Synedra_hyperborea (24.8) (2.79)	Protodinium_simplex (20.76) (5.62)	Chytriodinium_roseum (42.38) (3.22)	Pelagodinium_beii (25.62) (5.37)	Pseudo-nitzschia_sp. (61.56) (3.35)
7	Chaetoceros_neogracilis (24.28) (2.73)	Ephelota_mammillata (16.25) (4.4)	Fragilariopsis_sublineata (41.9) (3.18)	Gymnodinium_sp. (23.64) (4.95)	Gyrodinium_helveticum (50.55) (2.75)
8	TAGIRI1-lineage uc_sp. (20.92) (2.35)	Eukaryota uc (12.93) (3.5)	Woloszynskia_sp. (40.12) (3.05)	Stephanoecidae D uc_sp. (23.52) (4.93)	Strombidiidae_H uc_sp. (41.87) (2.28)
9	Raphid-pennate uc (19.08) (2.14)	Hypocoma_acinetarum (11.16) (3.02)	Pseudo-nitzschia_sp. (36.9) (2.8)	Azadinium uc (17.13) (3.59)	Gyrodinium_fusiforme (39.05) (2.13)
10	Odontella_aurita (17.48) (1.97)	MAST-9A uc_sp. (10.73) (2.91)	Dinophyceae uc (36.09) (2.74)	Prorocentrum_sp. (16.02) (3.36)	Chloroparvula_pacifica (37.55) (2.04)

Table S4: **F4 and HG-IV Top Abundance species of Class Bacillariophyta by sum off all ASV of this species:** For each cluster the top 10 species with their relative abundance and the portion of relative abundance of the cluster, calculated by summing up the ASVs relative abundance values for each of the both year according to the Class, are shown. The species that are referred in the text are colored light-gray.

	H-01 (NA)	H-02 (LL)	H-03 (LL)	H-04 (LL)	H-05 (HL)
1	Chaetoceros_socialis (31.04) (100.0)	Chaetoceros_sp. (7.68) (23.9)	Naviculales_sp. (26.29) (22.09)	Mediophyceae uc (1.97) (21.98)	Fragilariopsis_cylindrus (45.02) (19.72)
2	nan (0) (0.0)	Pleurosigma_intermedium (5.5) (17.1)	Bacillaria_paxillifer (18.41) (15.47)	Pinnularia uc (1.93) (21.46)	Grammonema_striatula (33.23) (14.56)
3	nan (0) (0.0)	Melosira_arctica (5.24) (16.29)	Tabularia_tabulata (17.35) (14.58)	Chaetoceros_gracilis (1.64) (18.29)	Chaetoceros_neogracilis (32.89) (14.41)
4	nan (0) (0.0)	Bacillariophyta uc uc (3.2) (9.96)	Synedra_hyperborea (12.13) (10.2)	Stauroneis_kriegeri (0.95) (10.6)	Bacillaria_paxillifer (18.6) (8.15)
5	nan (0) (0.0)	Fragilariopsis_sublineata (3.19) (9.93)	Pseudo-nitzschia_seriata (8.8) (7.39)	Pleurosigma_intermedium (0.95) (10.52)	Cylindrotheca_closterium (10.27) (4.5)
6	nan (0) (0.0)	Bacillaria_paxillifer (2.38) (7.39)	Attheya_septentrionalis (8.37) (7.04)	Pseudo-nitzschia uc (0.87) (9.65)	Chaetoceros_dichaeta (7.95) (3.48)
7	nan (0) (0.0)	Entomoneis_ornata (1.91) (5.95)	Navicula_sp. (8.35) (7.02)	Entomoneis_sp. (0.67) (7.49)	Odontella_aurita (7.81) (3.42)
8	nan (0) (0.0)	Araphid-pennate uc (1.55) (4.83)	Chaetoceros_sp. (6.67) (5.61)	nan (0) (0.0)	Raphid-pennate uc (7.78) (3.41)
9	nan (0) (0.0)	Grammonema_striatula (1.49) (4.64)	Amphora_sp. (5.82) (4.89)	nan (0) (0.0)	Navicula_radiosa (7.16) (3.13)
10	nan (0) (0.0)	nan (0) (0.0)	Araphid-pennate uc (5.03) (4.23)	nan (0) (0.0)	Araphid-pennate uc (6.16) (2.7)
	H-06 (HL)	H-07 (HL)	H-08 (HL)	H-09 (HL)	H-10 (HL)
1	Pseudo-nitzschia_sp. (61.75) (48.55)	Attheya_septentrionalis (15.6) (53.7)	Thalassiosira uc (90.48) (35.19)	Pseudo-nitzschia_sp. (114.88) (53.44)	Fragilariopsis uc (33.77) (31.65)
2	Mediophyceae uc (15.93) (12.52)	Mediophyceae uc (9.16) (31.54)	Fragilariopsis_sublineata (39.73) (15.45)	Raphid-pennate uc (44.32) (20.62)	Thalassiosira_sp. (12.75) (11.95)
3	Actinocyclus_curvatulus (14.8) (11.64)	Chaetoceros uc (4.29) (14.77)	Chaetoceros_danicus (31.4) (12.21)	Thalassiosira_sp. (23.49) (10.93)	Raphid-pennate uc (12.46) (11.68)
4	Minidiscus_trioculatus (11.77) (9.26)	nan (0) (0.0)	Chaetoceros_debilis_1 (29.21) (11.36)	Pseudo-nitzschia uc (19.0) (8.84)	Chaetoceros_brevis_3 (12.08) (11.32)
5	Pseudo-nitzschia uc (8.01) (6.3)	nan (0) (0.0)	Rhizosolenia_imbricata_var_shrubsolei (29.1) (11.32)	Chaetoceros uc (9.51) (4.42)	Pseudo-nitzschia_delicatissima (11.69) (10.96)
6	Raphid-pennate uc_sp. (4.87) (3.83)	nan (0) (0.0)	Eucampia_sp. (18.72) (7.28)	Rhizosolenia_formosa (3.76) (1.75)	Mediophyceae uc_sp. (9.07) (8.5)
7	Thalassiosira_sp. (3.95) (3.11)	nan (0) (0.0)	Chaetoceros_rostratus (6.97) (2.71)	nan (0) (0.0)	Cylindrotheca_closterium (4.96) (4.65)
8	Thalassiosira_antarctica (3.56) (2.8)	nan (0) (0.0)	Fragilariopsis_cylindrus (6.0) (2.33)	nan (0) (0.0)	Chaetoceros_dichaeta (4.41) (4.13)
9	Chaetoceros_gelidus (2.54) (2.0)	nan (0) (0.0)	Melosira_arctica (5.54) (2.16)	nan (0) (0.0)	Rhizosolenia_imbricata (2.88) (2.7)
10	nan (0) (0.0)	nan (0) (0.0)	nan (0) (0.0)	nan (0) (0.0)	Corethron_inerme (2.62) (2.46)
	F-01 (LL)	F-02 (LL)	F-03 (LL)	F-04 (LL)	F-05 (NA)
1	Leptocylindrus_minimus (2.54) (60.36)	Fragilariopsis_cylindrus (17.72) (30.59)	Chaetoceros_sp. (8.17) (50.38)	Bacillaria_paxillifer (1.88) (100.0)	Chaetoceros_debilis_1 (21.76) (40.91)
2	Stauroneis uc (1.67) (39.64)	Navicula_sp. (15.61) (26.95)	Melosira_arctica (6.11) (37.64)	nan (0) (0.0)	Tabularia_tabulata (12.22) (22.98)
3	nan (0) (0.0)	Naviculales_sp. (10.81) (18.67)	Conticribra_weissflogii (1.94) (11.98)	nan (0) (0.0)	Raphid-pennate uc_sp. (9.74) (18.3)
4	nan (0) (0.0)	Navicula uc (7.91) (13.65)	nan (0) (0.0)	nan (0) (0.0)	Araphid-pennate uc (5.93) (11.14)
5	nan (0) (0.0)	Thalassiosira_hispida (3.49) (6.02)	nan (0) (0.0)	nan (0) (0.0)	Mediophyceae uc (3.55) (6.67)
6	nan (0) (0.0)	Fragilariopsis_sublineata (2.38) (4.12)	nan (0) (0.0)	nan (0) (0.0)	nan (0) (0.0)
7	nan (0) (0.0)	nan (0) (0.0)	nan (0) (0.0)	nan (0) (0.0)	nan (0) (0.0)
8	nan (0) (0.0)	nan (0) (0.0)	nan (0) (0.0)	nan (0) (0.0)	nan (0) (0.0)
9	nan (0) (0.0)	nan (0) (0.0)	nan (0) (0.0)	nan (0) (0.0)	nan (0) (0.0)
10	nan (0) (0.0)	nan (0) (0.0)	nan (0) (0.0)	nan (0) (0.0)	nan (0) (0.0)
	F-06 (HL)	F-07 (NA)	F-08 (HL)	F-09 (NA)	F-10 (HL)
1	Grammonema_striatula (71.1) (26.09)	Thalassiosira_sp. (33.67) (41.0)	Thalassiosira uc (98.57) (27.64)	nan (0) (0.0)	Pseudo-nitzschia_sp. (61.56) (38.27)
2	Synedra_hyperborea (24.8) (9.1)	Raphid-pennate uc (33.19) (40.41)	Fragilariopsis uc (45.12) (12.65)	nan (0) (0.0)	Thalassiosira_sp. (21.4) (13.31)
3	Chaetoceros_neogracilis (24.28) (8.91)	Rhizosolenia_formosa (6.3) (7.67)	Fragilariopsis_sublineata (41.9) (11.75)	nan (0) (0.0)	Rhizosolenia_imbricata_var_shrubsolei (17.87) (11.11)
4	Raphid-pennate uc (19.08) (7.0)	Rhizosolenia_imbricata (3.37) (4.1)	Pseudo-nitzschia_sp. (36.9) (10.34)	nan (0) (0.0)	Actinocyclus_curvatulus (14.65) (9.1)
5	Odontella_aurita (17.48) (6.42)	Araphid-pennate uc (3.33) (4.05)	Chaetoceros_danicus (25.49) (7.15)	nan (0) (0.0)	Chaetoceros_brevis_3 (11.37) (7.07)
6	Minidiscus_trioculatus (16.87) (6.19)	Navicula_sp. (2.27) (2.77)	Mediophyceae uc (23.08) (6.47)	nan (0) (0.0)	Fragilariopsis_cylindrus (9.0) (5.6)
7	Nitzschia_sp. (14.76) (5.42)	nan (0) (0.0)	Bacillariophyta uc uc (18.55) (5.2)	nan (0) (0.0)	Raphid-pennate uc (6.74) (4.19)
8	Chaetoceros_socialis (13.61) (4.99)	nan (0) (0.0)	Chaetoceros uc (12.3) (3.45)	nan (0) (0.0)	Cylindrotheca_closterium (6.38) (3.97)
9	Bacillaria_paxillifer (10.22) (3.75)	nan (0) (0.0)	Eucampia_sp. (12.1) (3.39)	nan (0) (0.0)	Pseudo-nitzschia uc (5.24) (3.26)
10	Navicula_sp. (10.0) (3.67)	nan (0) (0.0)	Thalassiosira_sp. (8.23) (2.31)	nan (0) (0.0)	Corethron_hystrix (3.59) (2.23)

Table S5: **Mixed Layer Regime ASVs are reduced 2017 in comparison to 2018 in HG-IV:** The charred ASVs of F4 and HG-IV were grouped into meltwater regimes (MWR) and mixed layer regimes (MLR) based on their abundance ratio. Quotients of the location wise ratios of MWR and MLR were calculated for each year. In addition, MWR and MLR ASV Abundance ratios were calculated for each location between the years. For each year, the groups MWR and MLR were then tested using the KS test, whose distribution was greater/smaller using a one-sided test. These eight quotients were used to calculate the pairwise ratio of the MRW and MLW on different locations and years and normalized to 1.

**Name**	**Value**	**% shared ASV**	**% specific ASV**
Number of shared ASVs	583		
Number of ASVs with non Zero Abundance	559		
Number of MWR ASVs	94	0.1612	0.5839
Number of MLR ASVs	67	0.1149	0.4161
		**STD**	
q(F42017)	1.234558		
q(HG2017)	2.138047		
q(F42018)	0.6025356		
q(HG2018)	0.2653313		
median(p(F42017,HG-IV2017))	0.9245897	0.5691752	
median(p(F42018,HG-IV2018))	1.774752	5.317479	
median(t(F42017,HG-IV2017))	1.431774	4.379805	
median(t(F42018,HG-IV2018))	0.6382022	0.3910333	
**Kolmogorov-Smirnov test**	**D**	**p-value**	
p(F42017,HG-IV2017) vs t(F42017,HG-IV2017) (two sided)	0.3563036	6.25E-05	
p(F42017,HG-IV2017) vs t(F42017,HG-IV2017) (H_0 greater)	0.3563036	3.13E-05	
p(F42018,HG-IV2018) vs t(F42018,HG-IV2018) (two sided)	0.6592569	1.38E-14	
p(F42018,HG-IV2018) vs t(F42018,HG-IV2018) (H_0 less)	0.6592569	1.37E-14	
**Ratios**			
F4_2017_MWR_HP (polar)	0.9245897		
F4_2018_MWR_HP	0.45649254		
HG_2017_MWR_HP	1		
HG_2018_MWR_HP	0.31497868		
F4_2017_MLR_HP (atlant)	0.66966442		
F4_2018_MLR_HP	0.75761921		
HG_2017_MLR_HP	0.46771657		
HG_2018_MLR_HP	1.18711469		
quotient_of_mean_of_hg18_to_hg17	2.538107		
p(F42018,HG-IV2018) larger t(F42018,HG-IV2018)	2.78086161		
t(F42017,HG-IV2017) larger p(F42017,HG-IV2017)	1.54855067		
