# Supplementary figures and images for: Sea-ice melt determines seasonal phytoplankton dynamics and delimits the habitat of temperate Atlantic taxa as the Arctic Ocean atlantifies
Source: ISME Commun. 2024 Feb 27;4(1):ycae027. doi: 10.1093/ismeco/ycae027 (PMC10955684; doi:10.1093/ismeco/ycae027)

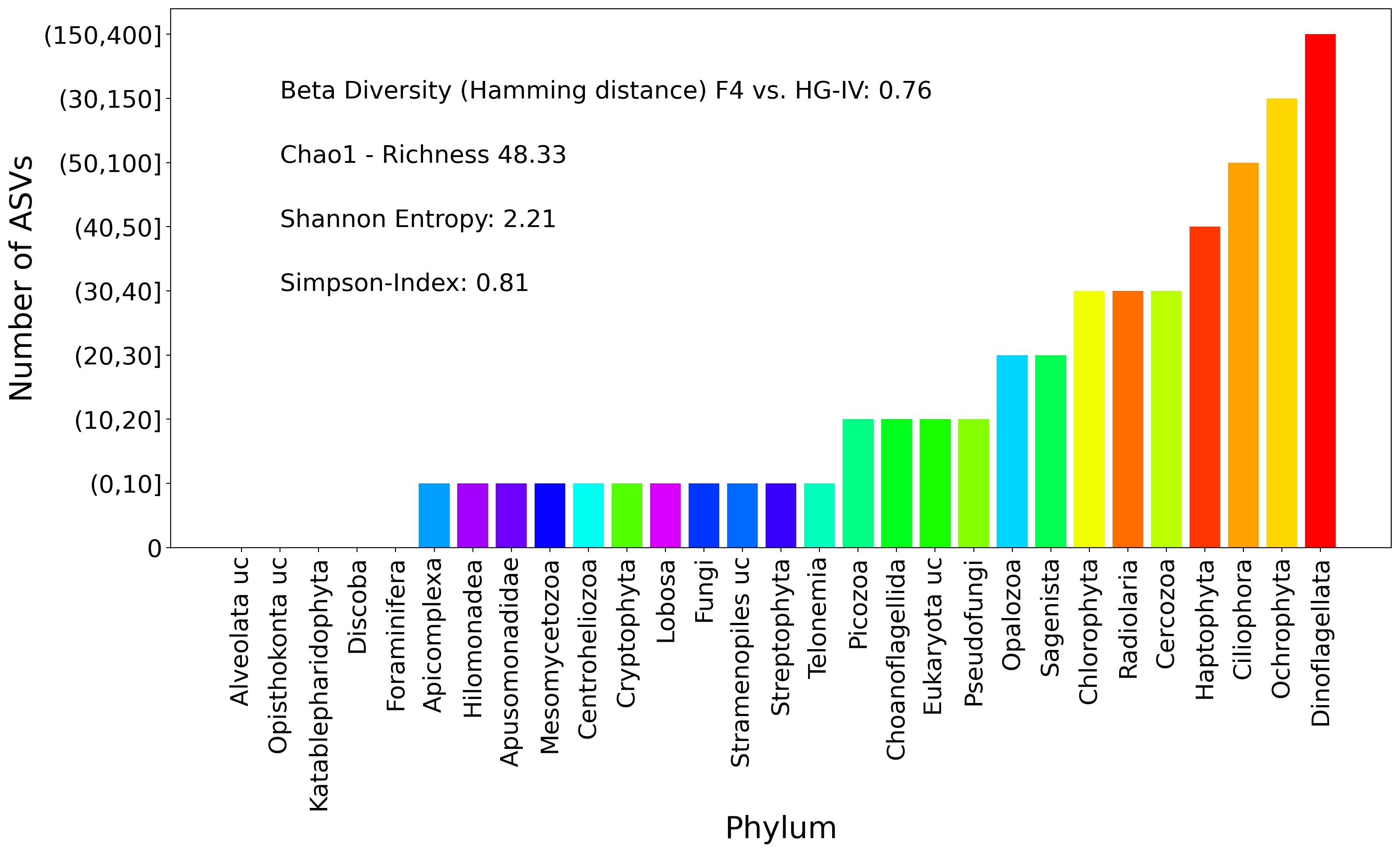

Supplement: FigS1_ycae027 [file figs1_ycae027.jpeg]

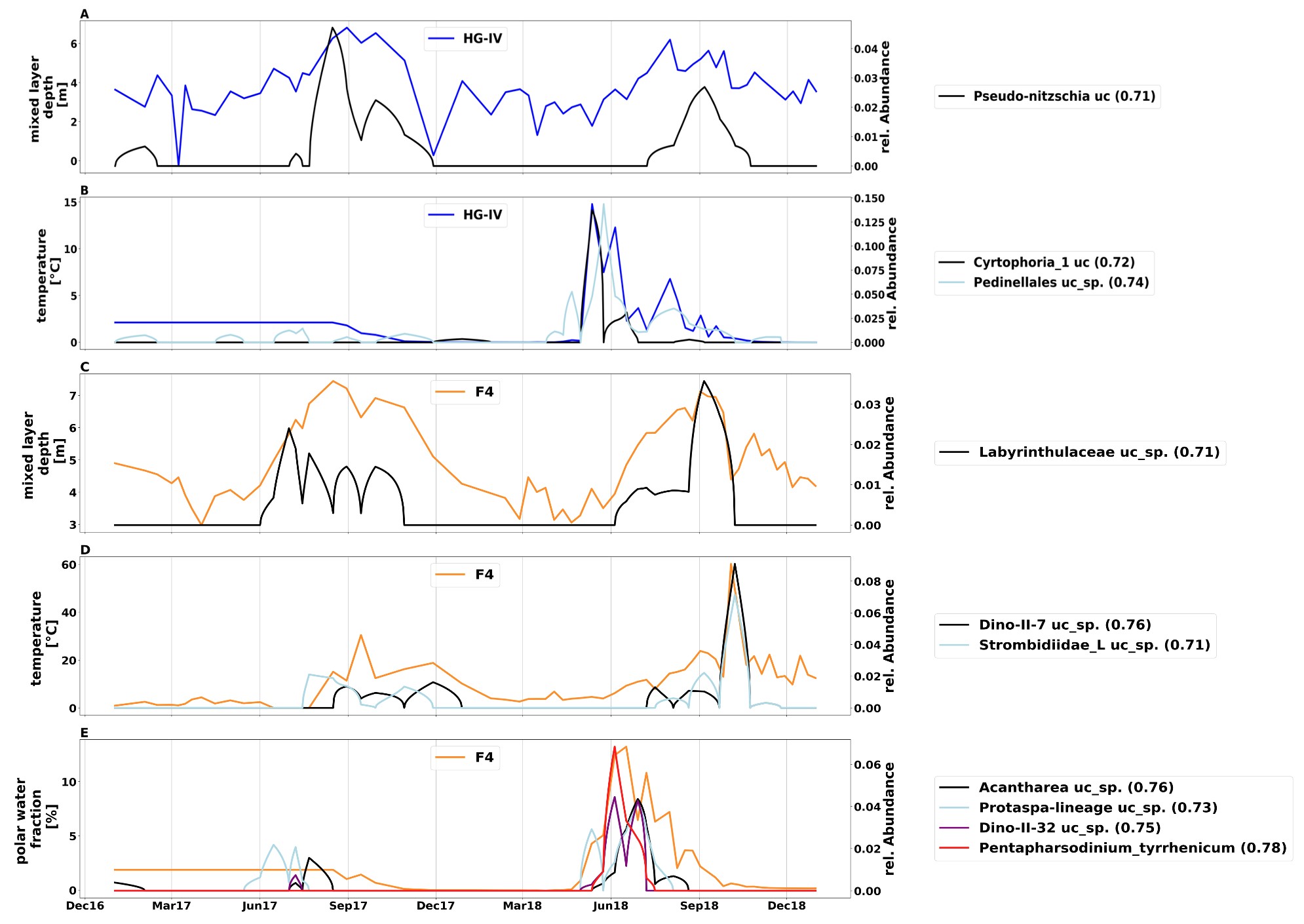

Supplement: FigS2_ycae027 [file figs2_ycae027.jpeg]

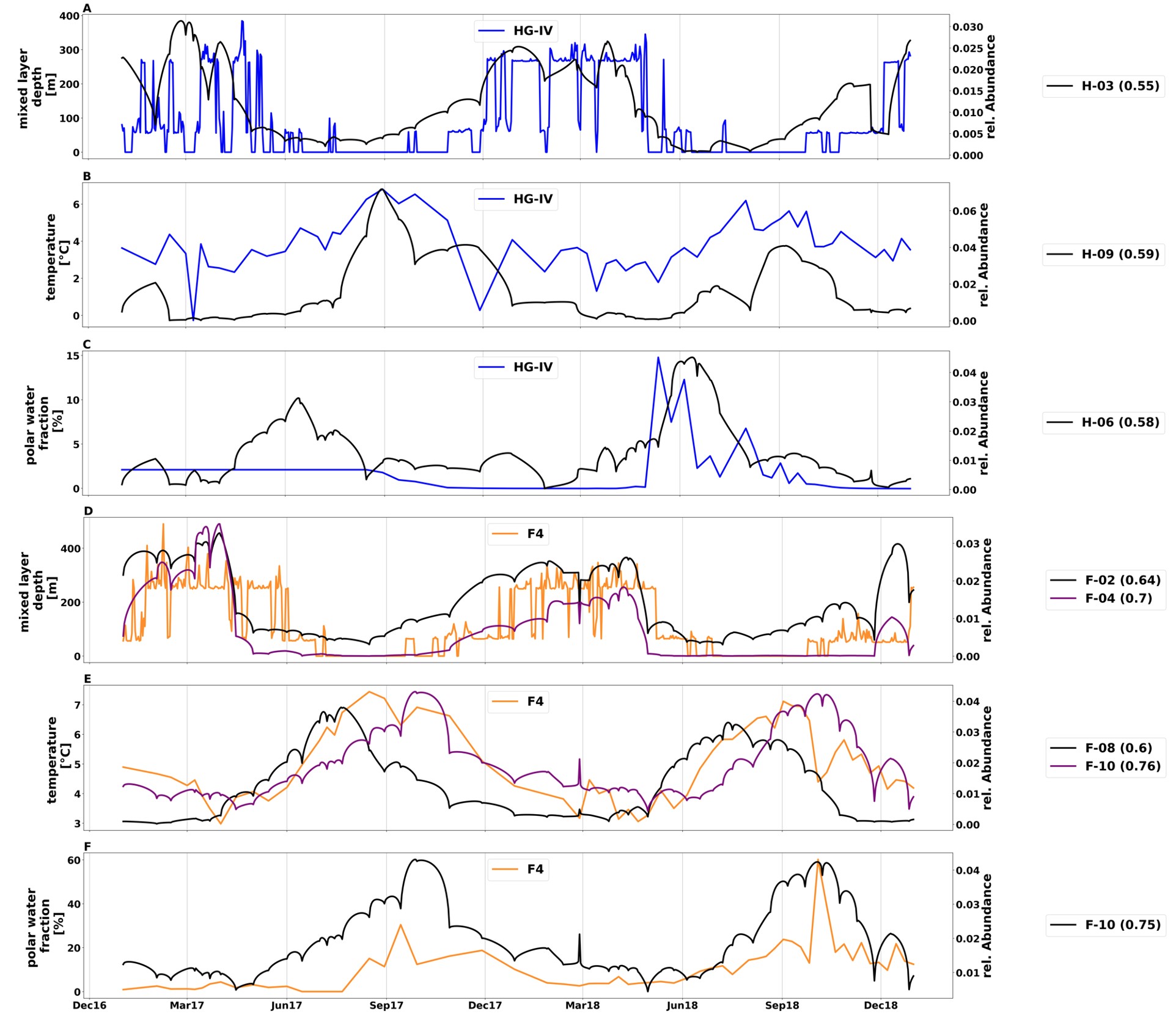

Supplement: FigS3_ycae027 [file figs3_ycae027.jpeg]

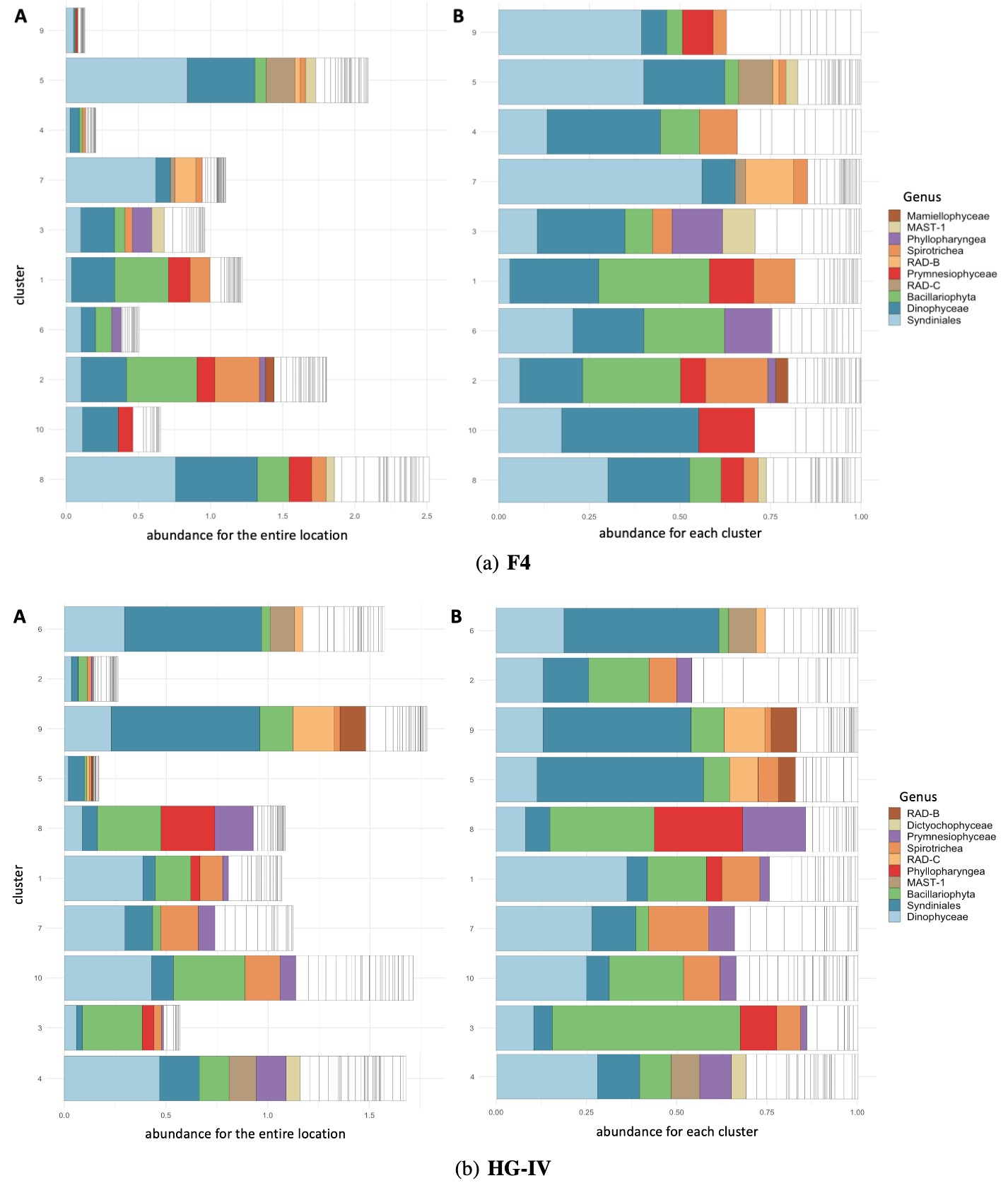

Supplement: FigS4_ycae027 [file figs4_ycae027.jpeg]

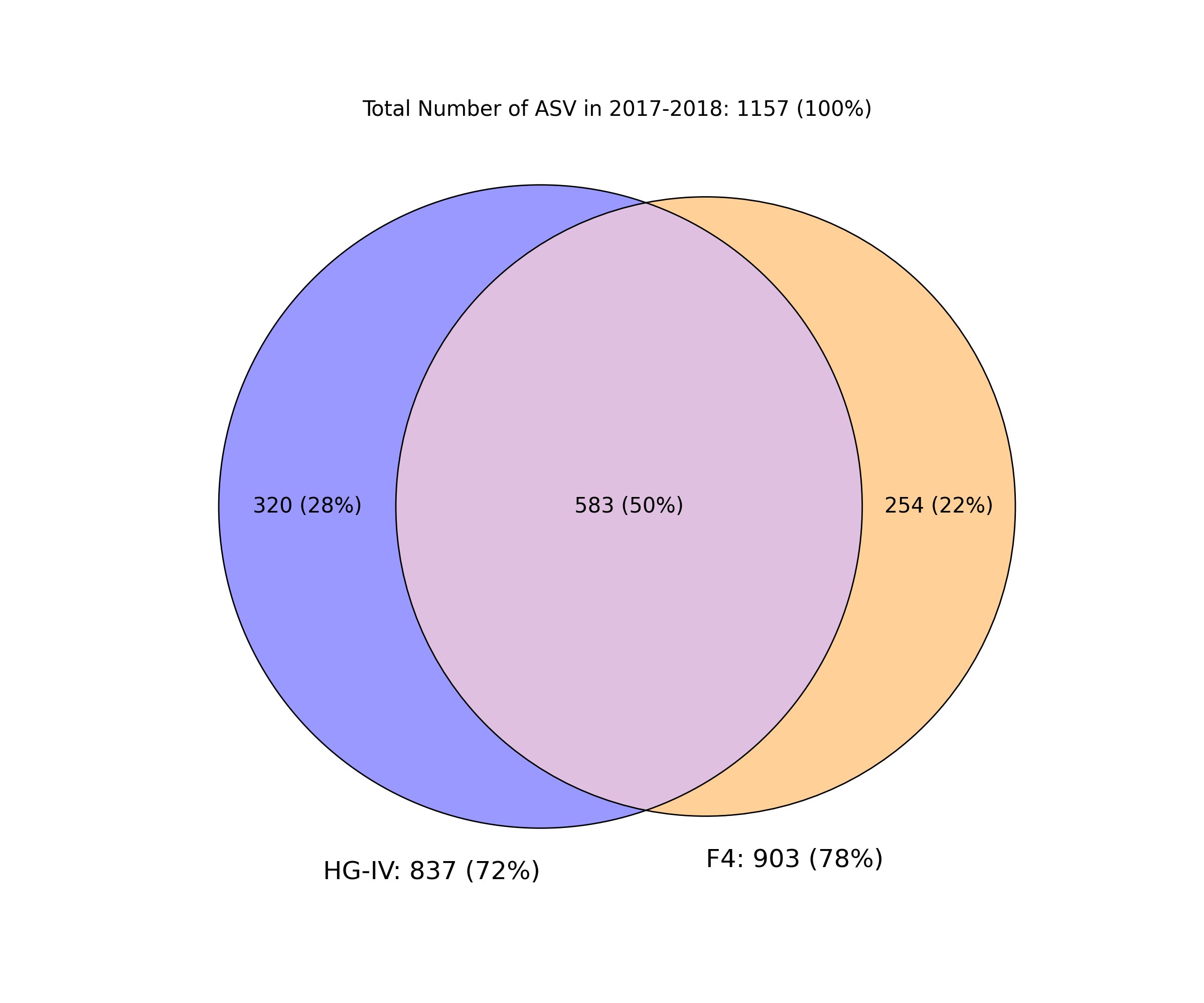

Supplement: FigS5_ycae027 [file figs5_ycae027.jpeg]
